# Variable Pathogenicity Determines Individual Lifespan in *Caenorhabditis elegans*

**DOI:** 10.1371/journal.pgen.1002047

**Published:** 2011-04-14

**Authors:** Adolfo Sánchez-Blanco, Stuart K. Kim

**Affiliations:** Department of Developmental Biology, Stanford University Medical Center, Stanford, California, United States of America; The University of North Carolina at Chapel Hill, United States of America

## Abstract

A common property of aging in all animals is that chronologically and genetically identical individuals age at different rates. To unveil mechanisms that influence aging variability, we identified markers of remaining lifespan for *Caenorhabditis elegans*. In transgenic lines, we expressed fluorescent reporter constructs from promoters of *C. elegans* genes whose expression change with age. The expression levels of aging markers in individual worms from a young synchronous population correlated with their remaining lifespan. We identified eight aging markers, with the superoxide dismutase gene *sod-3* expression being the best single predictor of remaining lifespan. Correlation with remaining lifespan became stronger if expression from two aging markers was monitored simultaneously, accounting for up to 49% of the variation in individual lifespan. Visualizing the physiological age of chronologically-identical individuals allowed us to show that a major source of lifespan variability is different pathogenicity from individual to individual and that the mechanism involves variable activation of the insulin-signaling pathway.

## Author Summary

One of the long-standing mysteries in aging is that some individuals die early whereas others die late. The age at which a specific individual will die is difficult or impossible to predict, and thus a fundamental aspect of aging in all animals is that it is stochastic. Aging stochasticity is particularly interesting in model organisms such as *C. elegans* because they are genetically inbred, can have the exact same chronological age, and can be grown under standard lab conditions. In this paper, we uncover a major mechanism underlying stochasticity in aging. To do this, we first developed a fluorescent aging marker (*sod-3::GFP*) whose expression declines with age and can be used to measure physiological age. In young animals, the level of expression of this fluorescent marker indicates which animals will live longer and which will die earlier. We used this fluorescent aging marker to show that variable pathogenicity from individual to individual is a major source of lifespan variability and that the mechanism involves variable activation of the insulin-signaling pathway.

## Introduction

A fundamental property of aging in all animals is stochasticity, which refers to the large and unpredictable variability in the lifespan of individuals in a population [1]. For example, human lifespan expectancy at birth in modern societies is about 78 years, but there is a large variability in the age of death of individuals as 29% die after age 85 and 27% die before age 65 (coefficient of variation is ∼0.2) [2]. Understanding the reasons that make some individuals die earlier than others would provide key insights about why some succumb to major killers such as infection, cardiovascular disease, cancer and stroke, whereas others do not. The causes of the variability in the aging process are poorly understood.

Aging stochasticity can best be studied in model organisms that are genetically identical and can be grown under controlled environmental conditions, such as the nematode *C. elegans*, a model organism with a normal lifespan of about two weeks [3]. For instance, an isogenic population of worms of the same age grown under identical conditions shows a great deal of individual variability in lifespan, with a coefficient of variation of ∼0.24 [4]. Individual animals appear to age at different rates, such that animals that are the same chronological age may have aged to different extents (younger or older) and have different physiological ages [5].

Several studies have characterized behavioral, morphological and molecular changes in old worms, These age-related changes include decline in locomotion [5], [6], decrease in the rate of pumping of the pharynx [7], and increase in an age-related pigment called lipofuscin [6], [8], [9]. Herndon et al. used electron microscopy to identify ultra-structural changes in different tissues and observed that the nervous system appeared to undergo much less dramatic changes during aging than the muscular system [5]. They also found a stochastic component for age-related decline in individuals. Muscle sarcomeres become damaged in old worms, and this damage can be visualized using a muscle myosin protein tagged with GFP (*MYO-3*::*GFP*) [5]. DNA microarray analysis has identified genes that change expression with age [10], [11], [12].

In some cases, the age-related changes have been shown to be markers of physiological, rather than just chronological age. Individual worms can age at different rates, such that some individuals may be physiologically older or younger than others even though they are the same chronological age. One test of whether an age-related change is a marker of physiological age is to see if it predicts remaining lifespan of individuals of the same chronological age. For instance, high lipofucsin levels in moderately aged worms correlates with short lifespans. Another example is expression of *hsp-16.2* [13]. Heat shock protects cells and extends lifespan by inducing a number of heat shock response genes, including *hsp-16.2*. Following heat shock, levels of expression of *hsp-16.2* correlate with remaining lifespan of individual worms [13]. However, it is not clear whether differences between individuals occur naturally or are due to the heat shock treatment.

We wanted to understand the mechanisms underlying aging stochasticity. To pursue this goal we first identified *C. elegans* genes whose expression in individual worms was predictive of their remaining lifespan. We used *sod-3*, one of the genes whose expression was predictive of remaining lifespan, to investigate mechanisms underlying variable aging in individuals. Our results indicate that pathogenicity from *Escherichia coli* used as food is a major source of lifespan variability due to variable activation of the insulin-signaling pathway.

## Results

### Identification of molecular markers predictive of remaining lifespan

**Figure 1:**
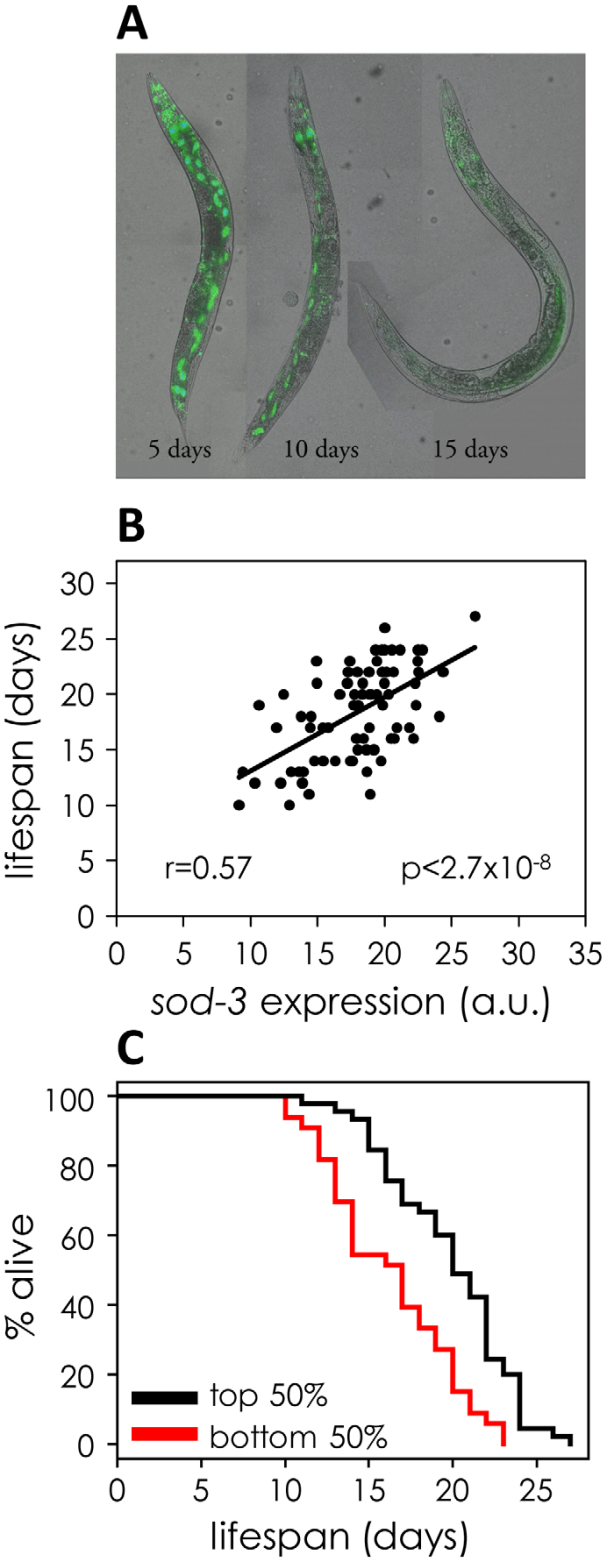
*sod-3* expression correlates with remaining lifespan. (A) *sod-3::GFP* expression decreases in old worms. Shown are images of three worms at 5, 10 and 15 days of adulthood expressing extrachromosomal *sod-3::GFP*. (B) Correlation of *sod-3* expression at day 9 with remaining lifespan (n = 78). x-axis shows mCherry fluorescence in arbitrary units. y-axis shows lifespan in days. The correlation between expression and lifespan is r = 0.57, with a p-value<2.7×10^−8^. (C) Worms with *sod-3* expression in the top 50% at day 9 live 22% longer than those with expression in the bottom 50% (p = 5.76×10^−5^ (log rank)). y-axis shows percentage of worms alive. x-axis shows lifespan in days. Data are from the same worms as in (B).

We wanted to understand the underlying factors causing aging variability in *C. elegans*. To achieve this goal, our strategy was to find markers that not only change with age, but that are predictive of remaining lifespan of individuals in a synchronous population. A marker whose expression is connected to an age-related process that ultimately limits lifespan should reveal which individuals will die early or live long. We tested eight fluorescent reporters corresponding to genes that change expression with age (Figure 1A, Figure S1, Table 1). For each fluorescent reporter we measured fluorescence of individual worms at the point when they had lived ∼50% of their mean lifespan. We then recorded the remaining lifespan of each individual, and compared the level of fluorescence expression with their lifespan. For a *sod-3::mCherry* reporter (fluorescent mCherry protein driven by the superoxide dismutase-3 promoter), we found that mCherry abundance showed a Pearson correlation of 0.57 with lifespan, thus explaining 32% of the variation in lifespan between individuals (Figure 1B). We separated the worms into two groups according to the abundance of *sod-3* mCherry, and found that worms with more *sod-3* expression lived on average 22% longer than their siblings with less expression (Figure 1C). Besides the *sod-3::mCherry* reporter, we were able to correlate fluorescence abundance and remaining lifespan using two other *sod-3* reporters: a transcriptional GFP reporter containing multiple integrated copies [14] and a transcriptional reporter expressing histone H2B fused to GFP on an extrachomosomal array [10]. The remaining seven reporters showed a Pearson correlation between fluorescent intensity and remaining lifespan, ranging from 0.35 to 0.51 (Table 1). In summary, we found a correlation between gene expression and lifespan for eight genes whose expression changes with age. For the remainder of this paper, we focus our attention on *sod-3*, which is the best marker of physiological age of those tested.

**Table 1:** Correlation of fluorescent markers with remaining lifespan.

gene name	expression trend during aging	n	r^a^	p^b^
*sod-3* c	↓	78	0.57	2.7×10^−8^
*ugt-9*	↓	74	0.50	3.0×10^−6^
*elt-3*	↓	77	0.40	1.6×10^−4^
*daf-16*	↓	78	0.46	9.2×10^−6^
*unc-54*	↓	79	0.51	8.0×10^−7^
*myo-3*	↓	80	0.44	1.9×10^−5^
*C26B9.5*	↓	80	0.38	2.3×10^−4^
*pha-4*	↑	80	-0.34	8.2×10^−4^
*mif-2*	← →	78	0.15	9.7×10^−2^
*his-72*	← →	72	0.28	1.5×10^−2^
*C08B11.3*	← →	71	0.07	0.28
*sur-5*	← →	77	0.30	3.7×10^−3^
lipofuscin	↑	79	0.02	0.42

a correlation coefficient between gene expression and lifespan.

b one tailed p-value for correlation significance.

c *sod-3::mCherry*.

We examined *sod-3* expression at different ages to find the optimal time for using *sod-3* expression as an aging marker. At ages earlier than day 9, we found that *sod-3* expression can still correlate with remaining lifespan in individual worms, although not as well as in 9 day old worms (∼50% of mean lifespan) (Table S1). At ages later than day 9, *sod-3* expression is not as useful as a molecular marker because worms begin to show overt signs of aging such as decreased locomotion, decreased pharyngeal pumping and vacuolar appearance. Next, we analyzed *sod-3::mCherry* expression in the same worm at different ages in a longitudinal assay (Figure S2). This experiment revealed the age-related downward slope in expression of *sod-3* for each individual worm, in addition to levels of *sod-3* expression at day 9. However, using linear regression, we found that the age-related slope of *sod-3* expression did not improve the prediction of remaining lifespan beyond the prediction made using *sod-3* expression at 9 days of age alone (p<0.21). In summary, we measured *sod-3* expression in a variety of ways and found that expression at day 9 was most informative for predicting remaining lifespan in individual worms.

*sod-3* activity might directly extend longevity by itself, or it might be a marker for other processes in the worm that affect lifespan. *sod-3* null mutants are known to have wild-type lifespans [15], [16], [17]. We created transgenic lines containing many copies of wild-type *sod-3*, and found no differences in lifespan compared to controls (Figure S3). These results indicate that *sod-3* activity does not extend lifespan *per se*, but rather that *sod-3* expression is a reporter of remaining lifespan.

As a control, we selected four genes at random from the genome (*mif-2*, *his-72*, *C08B11.3*, and *sur-5*), measured their expression level in individual worms when they had lived ∼50% of their mean lifespan, and found that none correlated with remaining lifespan as well as the eight age-regulated genes (Table 1). Thus, not every gene can serve as a marker for aging. Therefore, the correlation between expression of age-related genes and remaining lifespan is not due to a general decrease in gene expression during aging.

Finally, we tested whether amounts of the age-related pigment lipofuscin correlated with remaining lifespan in middle-aged worms. Lipofuscin is composed of a set of fluorescent breakdown products that accumulate in the gut with age (Figure S1) [3], [8]. Previous studies have shown that lipofuscin levels correlate with remaining lifespan; specifically, in a chronologically old population, there is a small amount of worms that can barely move (Class C), and these worms have high levels of lipofucsin and short remaining lifetimes [9]. We tested whether lipofucsin autofluorescence correlates with remaining lifespan in middle-aged worms (day 9), before overt signs of aging appear. We found no correlation between lipofucsin levels and remaining lifespan (Table 1).

### Molecular and environmental sources of lifespan variability

**Figure 2:**
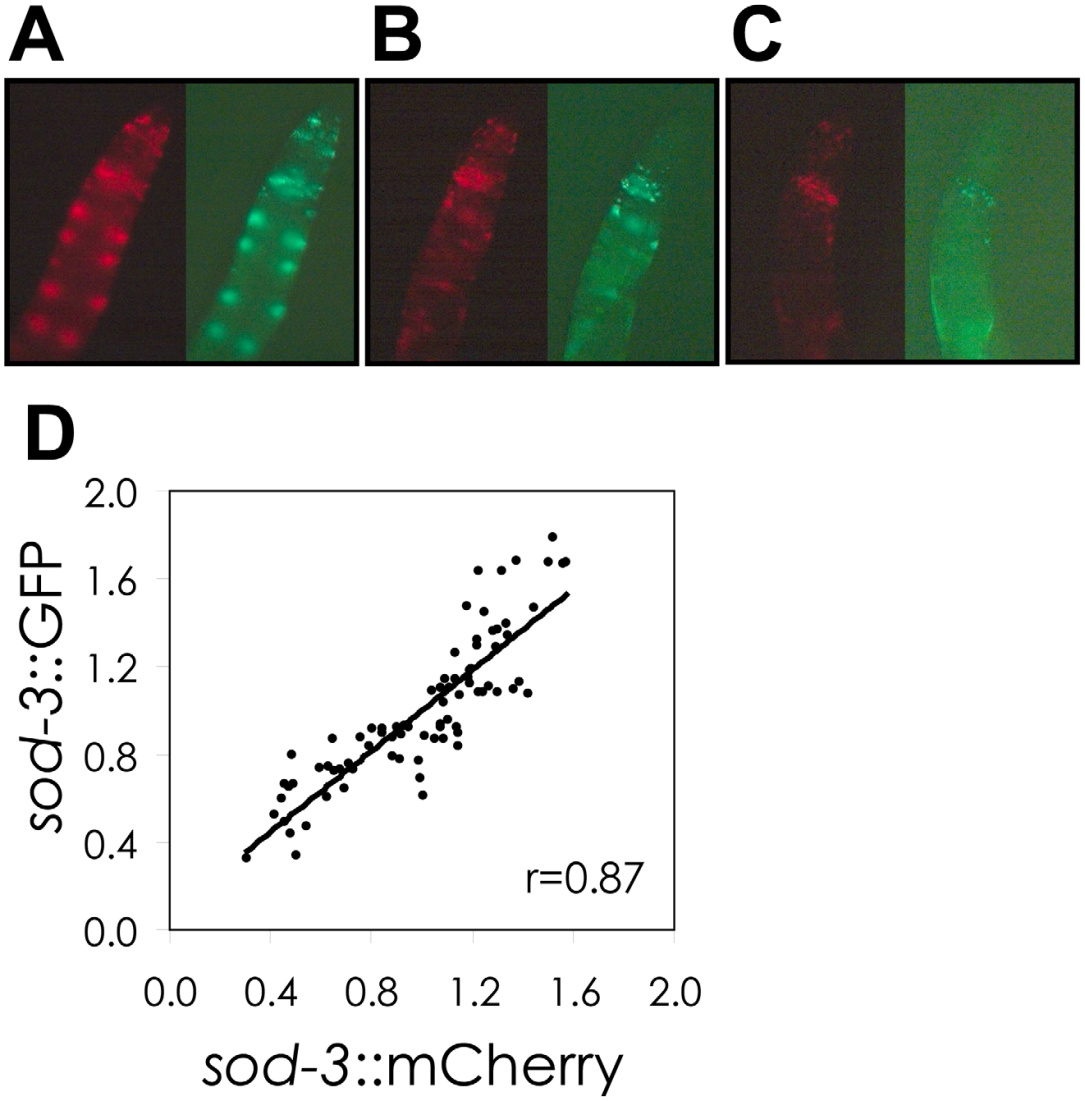
Correlation of *sod-3::GFP* and *sod-3::mCherry* reporter expression. (A–C), Shown are GFP and mCherry images of the anterior portion of adult hermaphrodites at day 8 of adulthood. The individual worms show high (A), medium (B), and low (C) abundance of GFP and mCherry. (D) Scatterplot comparing *sod-3* GFP and mCherry expression for 8 day old worms. Each dot represents expression from an individual worm. Data are from 80 *sod-3::mCherry*/extrachromosomal *sod-3::GFP* individuals. Correlation between relative levels of GFP and mCherry expression is r = 0.87.

There are many potential sources that could lead to variable abundance of *sod-3* between worms, including sources that are intrinsic (variable activity of transcription factors at the *sod-3* promoter) or extrinsic (variable effects from the environment). To begin to characterize the types of mechanisms that could be responsible for the correlation between variable lifespan and variable *sod-3* expression, we examined whether expression of two different *sod-3* reporters fluctuated either together or independently in individual worms from a synchronous population. We created a transgenic line expressing *sod-3::GFP* and *sod-3::mCherry*, and compared the fluorescence intensity of GFP and mCherry in individual worms. There was a high degree of correlation (r = 0.87) between *sod-3* expression from the GFP and mCherry reporters (Figure 2). This indicates that variability in *sod-3* expression is caused by a mechanism that is variable from worm to worm, but has similar effects on both reporter genes within an individual worm.

We tested several environmental factors that could vary between worms and be responsible for fluctuation in expression of *sod-3* and stochasticity in lifespan.

**Figure 3:**
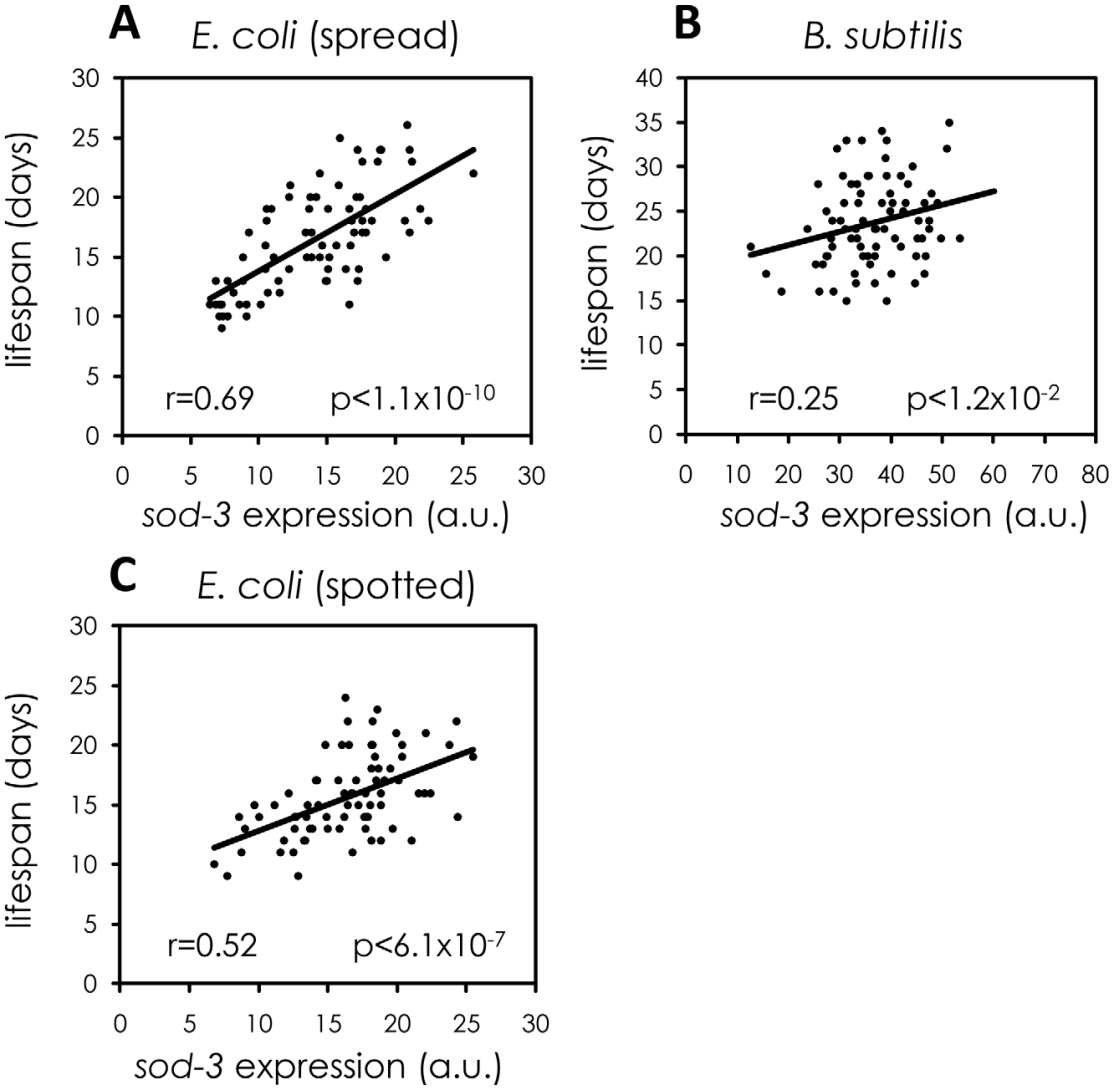
Variable pathogenicity as a source of variability in *sod-3* expression and lifespan. Shown are scatterplots correlating *sod-3::GFP* expression to their remaining lifespan. x-axis shows expression levels in arbitrary units. y-axis shows lifespan in days. The Pearson correlation between expression and remaining lifespan and p-value are shown in each plot. (A) *sod-3::GFP* expression in worms (n = 78) grown on plates with evenly spread *E. coli*. *sod-3::GFP* expression was measured at day 8 (49% of their mean lifespan). (B) *sod-3::GFP* expression in worms (n = 79) maintained on *B. subtilis*. *sod-3::GFP* expression was measured at day 12 (50% of their mean lifespan). (C) *sod-3::GFP* expression from worms (n = 79) grown on plates with a spot of *E. coli* in the middle. *sod-3::GFP* expression was measured at day 8 of adulthood (50% of their mean lifespan).

One possibility is that variability in individual lifespan might result from differences in feeding on bacteria provided as food on the culture plate, which could lead to heterogeneity in caloric restriction. However, *sod-3::GFP* fluorescence from worms grown on plates with an even lawn of *E. coli* correlated with remaining lifespan as well or better than that of worms grown on plates on which the supply of *E. coli* was restricted to one small spot (Figure 3A and 3C), indicating that heterogeneity in caloric restriction does not contribute to the link between variability in *sod-3* expression and lifespan.

**Figure 4:**
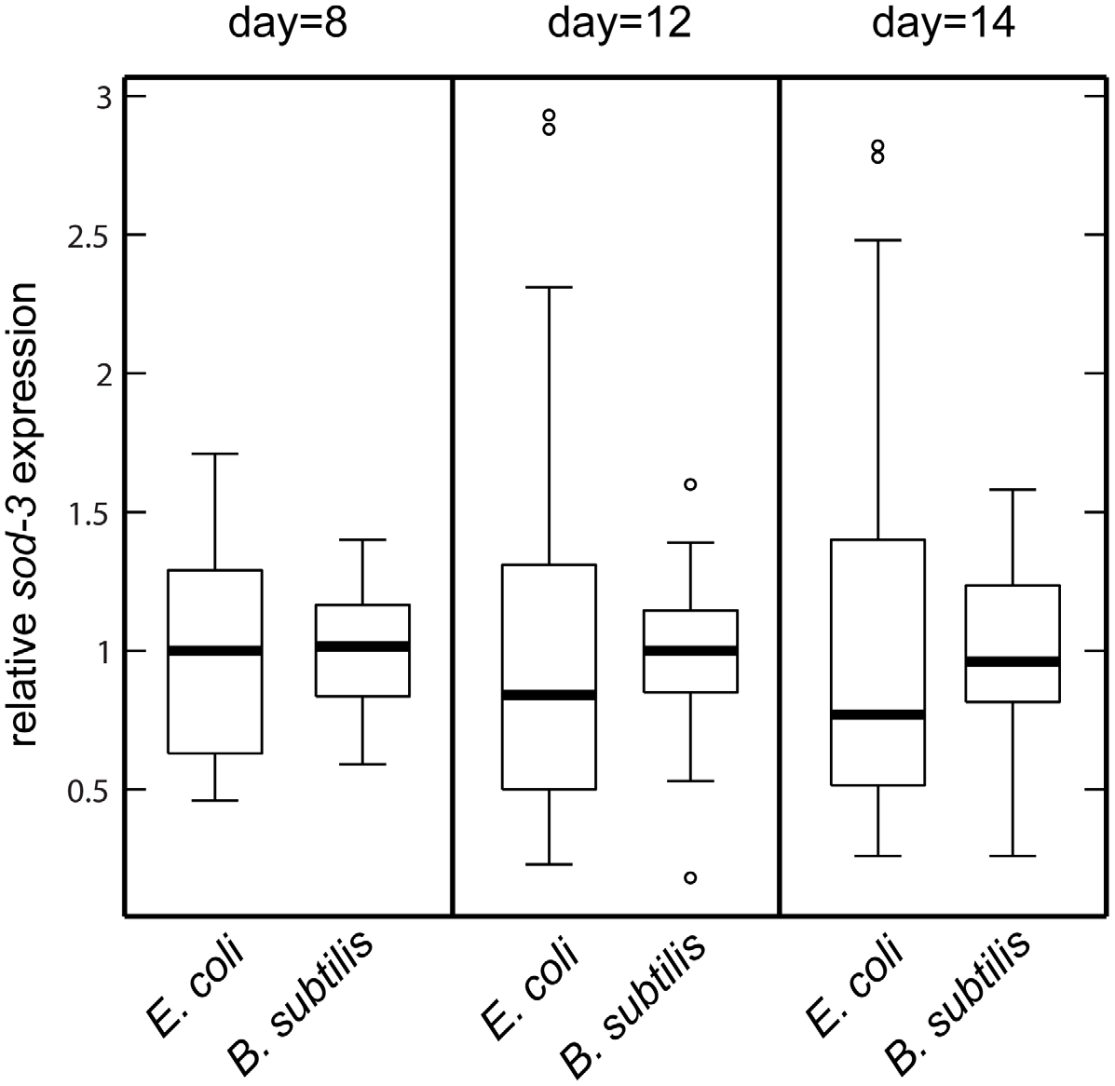
*sod-3* expression variability is lower for worms fed *B. subtilis* compared to worms fed *E. coli*. Shown are boxplot distributions for *sod-3*::GFP expression in worms maintained on *E. coli* or *B. subtilis* at 20°C at different ages. The boxes define the interquartile range and the thick line is the median. Bars represent the expression range. n = 60 in each group.

Another possibility is that variable pathogenicity from bacterial food could lead to variable *sod-3* expression and lifespan. *E. coli*, the common diet for worms, is mildly pathogenic whereas *Bacillus subtilis* is not pathogenic [18]. Accordingly, worms fed *B. subtilis* live longer than worms grown on *E. coli* [19], [20] (Figure S4). We tested whether variable pathogenicity could lead to individual differences in *sod-3* expression and lifespan in two ways. First, we examined whether growing worms on *B. subtilis* rather than *E. coli* reduced variability in *sod-3* expression. We cultured worms on *E. coli* or on *B. subtilis* for 8, 12, and 14 days and calculated variability in *sod-3* abundance (defined as Standard Deviation/mean) at each age. We found that the variability in *sod-3* expression was lower in worms fed *B. subtilis* than it was in worms fed *E. coli* at all ages (Figure 4 and Table S2). Second, we showed that the correlation between abundance of *sod-3::*GFP and remaining lifespan was considerably lower for worms maintained on *B. subtilis* than for worms grown on *E. coli* when worms had lived 50% of their mean lifespan (Figure 3A and 3B). Besides *B. subtilis*, we obtained similar results using two other non-pathogenic diets, UV-killed *E. coli* and *Caulobacter crescentus*. Specifically, worms fed these diets lived longer than *E. coli* fed worms (Figure S4), and the correlation between *sod-3* abundance and remaining lifespan was lower for worms fed these non-pathogenic diets than for *E. coli* fed worms when worms had lived 50% of their mean lifespan (Figure S5). Thus, these results suggest that variable pathogenicity from individual to individual may cause variation in abundance of *sod-3* and lifespan variability.

**Figure 5:**
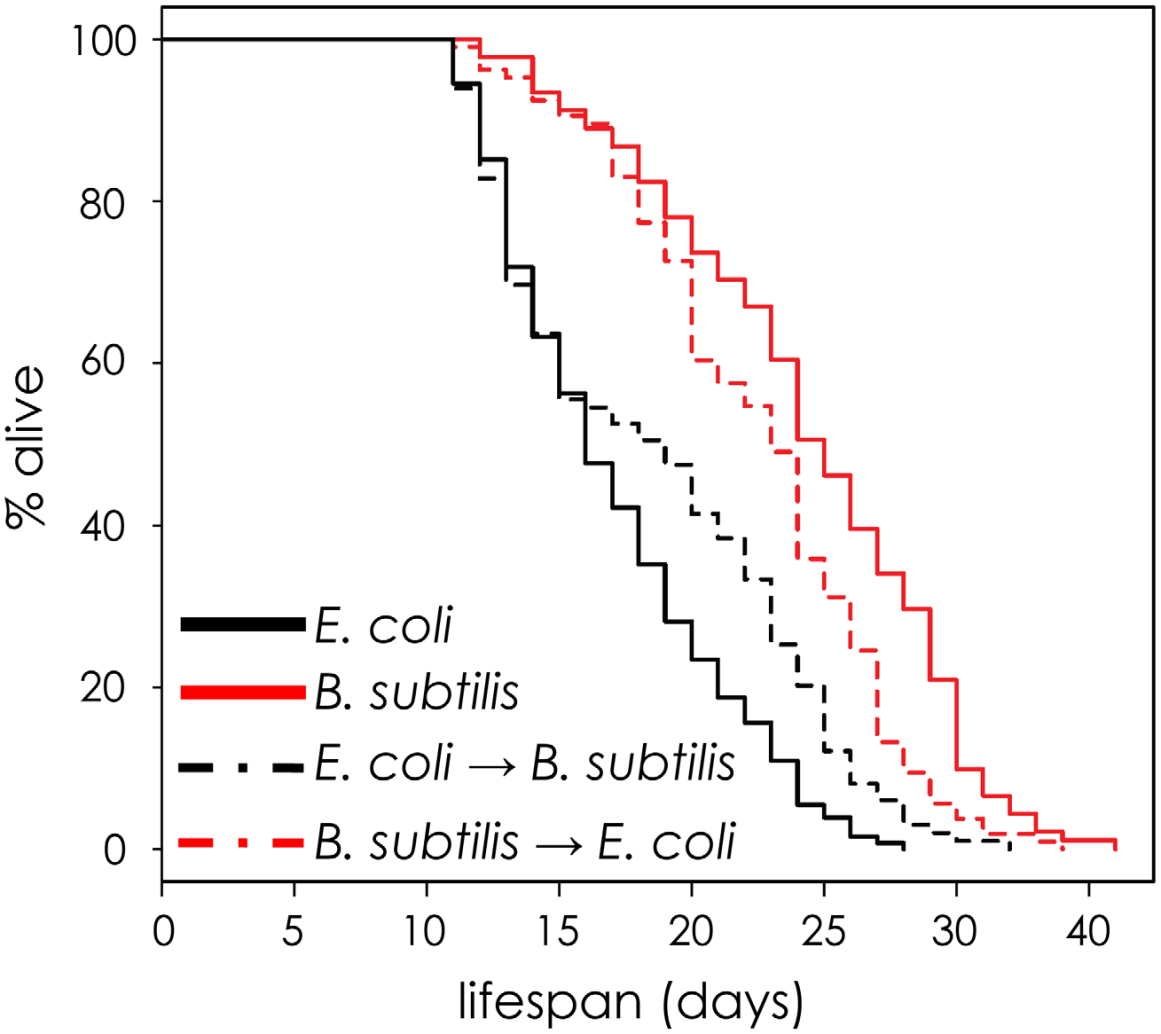
The effect of *E. coli* pathogenicity persists after worms are shifted to *B. subtilis*. Lifespan curves for worms maintained on one type of bacteria (*E. coli* or *B. subtilis*) and then shifted to the other bacteria at day 8. All lifespans were done at 20°C. y-axis indicates % of worms that are alive. x-axis indicates day of adulthood.

The finding that the amount of *sod-3* expression present in a middle-aged worm is correlated with its remaining lifespan indicates that events have occurred that affect its future aging trajectory. If so, feeding a worm either *E. coli* or *B. subtilis* should have greatest effect when it is young rather than when it is old. To test this, we fed worms one type of bacteria (*E. coli* or *B. subtilis*) when they were young and then shifted them to the other type of bacteria at day 8 of adulthood. Young worms fed *E. coli* had short lifespans, no matter what they ate when they were old. Conversely, young worms fed *B. subtilis* had long lifespans no matter what they ate when they were old (Figure 5 and Table S3). This result indicates that pathogenicity or some other factor associated with *E. coli* initiates changes in young worms that affect their time of death later on.

**Figure 6:**
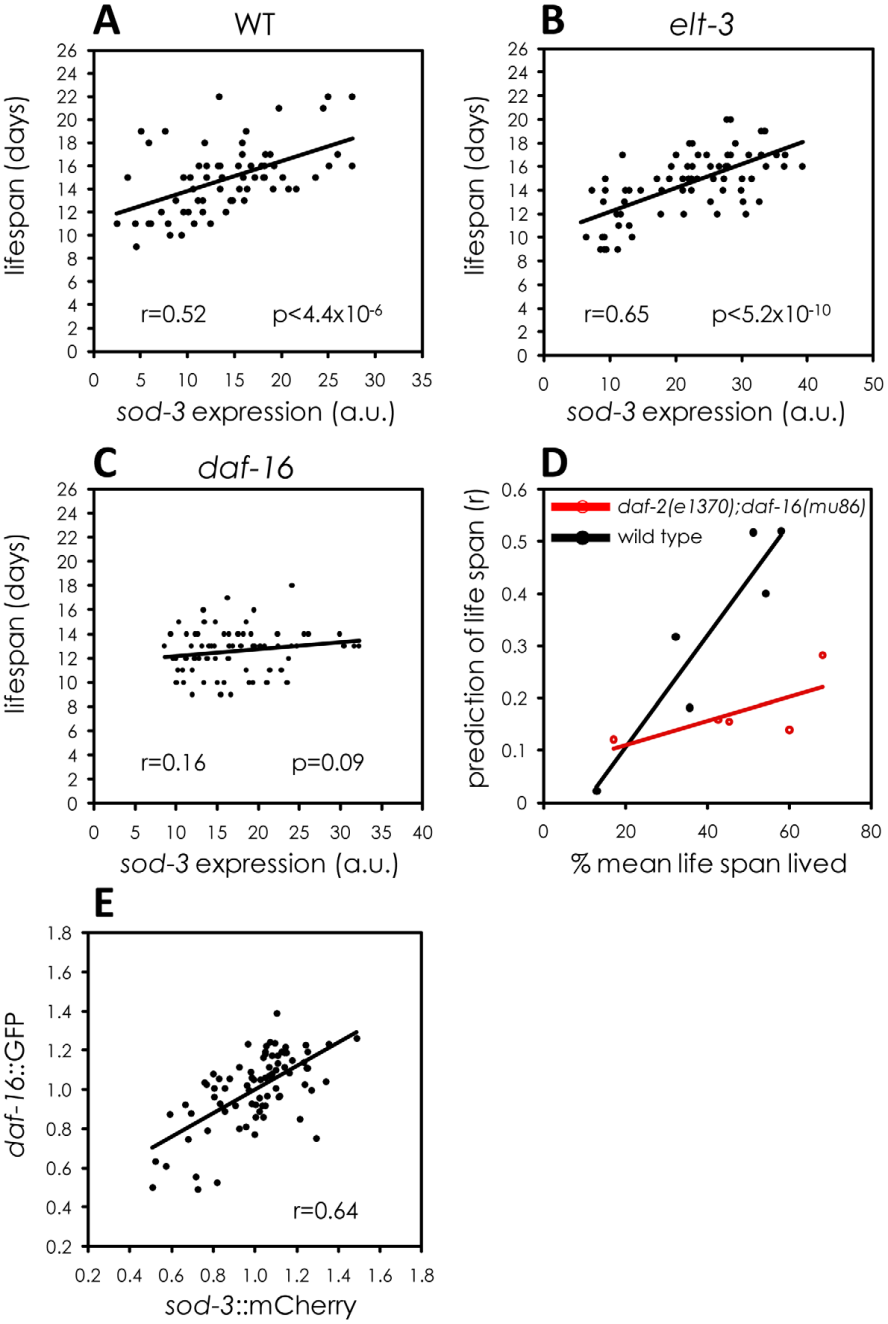
Variable *daf-16* activity as a source of *sod-3* expression and lifespan variability. (A–C) Shown are scatterplots comparing *sod-3::GFP* expression with their remaining lifespan. x-axis shows expression levels in arbitrary units. y-axis shows lifespan in days. The Pearson correlation between expression and remaining lifespan and p-value are shown in each plot. *sod-3::GFP* expression and lifespan are shown for (A) *daf-16(+);elt-3(+)* 9 day old worms (n = 68). (B) *elt-3(vp1)* 8 day old worms (n = 70). (C) *daf-16(mu86)* 8 day old worms (n = 76). (D) Shown is a scatterplot that summarizes data from several assays. Each point represents an experiment comparing *sod-3*::*GFP* expression with remaining lifespan. The y-axis shows the correlation coefficient and the x-axis shows the age of the worms when *sod-3* expression was measured. Black indicates worms that were *daf-2(+);daf-16(+)* and red indicates worms that were *daf-2(e1370);daf-16(mu86)* mutants. (E) Correlation between *daf-16::*GFP and *sod-3::mCherry* relative abundance in individual worms at day 9 (n = 78).

Pathogenicity might affect *sod-3* abundance through regulation of the insulin-like signaling pathway because this pathway mediates response to pathogen infection [19], [21], [22]. Furthermore, the terminal step in the insulin-like signaling pathway is *daf-16*, which encodes a FOXO transcription factor that directly regulates *sod-3* expression [23]. Fluctuation in the activity of *daf-16* FOXO could be a source of lifespan stochasticity in individual worms. We tested this possibility using four approaches. First, we showed that a *daf-16* null mutation eliminated the correlation between *sod-3* expression and lifespan (Figure 6C and Figure S6). The *daf-16* null mutation also prevents *sod-3* from acting as an aging marker at younger ages (Figure 6D). The lack of correlation between *sod-3* expression and lifespan in *daf-16* null mutants is not simply because *sod-3* is less abundant. *sod-3::GFP* was also less abundant in mutant worms for *elt-3* GATA (Figure S7), another transcription factor that regulates *sod-3* expression [10], but *sod-3* abundance in *elt-3* GATA mutants still correlated with remaining lifespan (Figure 6B).

Second, we tested whether the specific amount of DAF-16 in an individual worm led to corresponding changes in *sod-3* expression. The insulin-like signaling pathway controls activity of DAF-16 FOXO primarily by affecting protein phosphorylation, which controls nuclear versus cytoplasmic localization [24], [25]. However, it is possible that insulin-signaling may also affect the levels of total DAF-16 protein in the cell, which can be measured using a *daf-16::GFP* translational reporter. We constructed a strain that expressed a fusion of GFP with DAF-16 [24] as well as mCherry under the control of the *sod-3* promoter. We measured expression of GFP and mCherry in individual worms. Accumulation of these reporter proteins in individual worms was highly correlated (Figure 6E). This result is consistent with the model that DAF-16 is a direct regulator of *sod-3* expression.

Third, since *daf-16* is a direct regulator of *sod-3* expression [14] and expression of these genes is highly correlated with one another, one might expect that independently measuring expression of these two genes in the same worm would be either partially or wholly redundant. To test this, we evaluated a regression model that included expression of both genes to find out if expression of both genes together was more informative about remaining lifespan than expression of *sod-3* alone. We found that measuring expression of both genes together or *sod-3* alone showed little difference in predicting remaining lifespan (p = 0.26) (Table 2). This shows redundancy in lifespan information from *daf-16* and *sod-3* expression.

**Table 2:** Correlation of two fluorescent markers with remaining lifespan.

mCherry marker	GFP marker	n	correlation^a^	mCherry (r)^b^	GFP (r)^c^	mCherry/GFP (r)^d^	p^e^
*sod-3*	*DAF-16*	78	0.64	0.57	0.46	0.58	0.26
*sod-3*	*sod-3*	80	0.87	0.64	0.58	0.64	0.59
*sod-3*	*elt-3*	77	0.40	0.57	0.40	0.60	0.04
*sod-3*	*ugt-9*	74	0.44	0.60	0.50	0.66	4.1×10^−3^
*sod-3*	*myo-3*	70	0.69	0.52	0.54	0.58	0.01
*unc-54*	*sod-3*	72	0.61	0.53	0.59	0.63	0.02
*C26B9.5*	*sod-3*	75	0.08	0.36	0.60	0.67	4.1×10^−4^
*pha-4*	*sod-3*	80	−0.17	−0.37	0.64	0.70	1.4×10^−3^

a correlation (r) between mCherry and GFP marker expression.

b lifespan prediction (r) by mCherry marker.

c lifespan prediction (r) by GFP marker.

d lifespan prediction (r) by both markers combined.

e p-value comparing the regression model using *sod-3* expression alone to the regression model using both markers.

Fourth, we tested whether pathogenicity from *E. coli* used as food induces expression of *daf-16*. We fed worms either *E. coli* or non-pathogenic *B. subtilis* and measured the level of expression of DAF-16 during aging. We found that expression of *DAF-16::GFP* was significantly higher for worms fed *E. coli* than for worms fed *B. subtilis* at days two, five, and eight of adulthood (Figure S8). In summary, results from all four experiments indicate that fluctuations in DAF-16 FOXO activity account for much of the individual variability in *sod*-3 expression and lifespan in individual worms.

### The intestine is a major tissue determining variable individual lifespan

**Figure 7:**
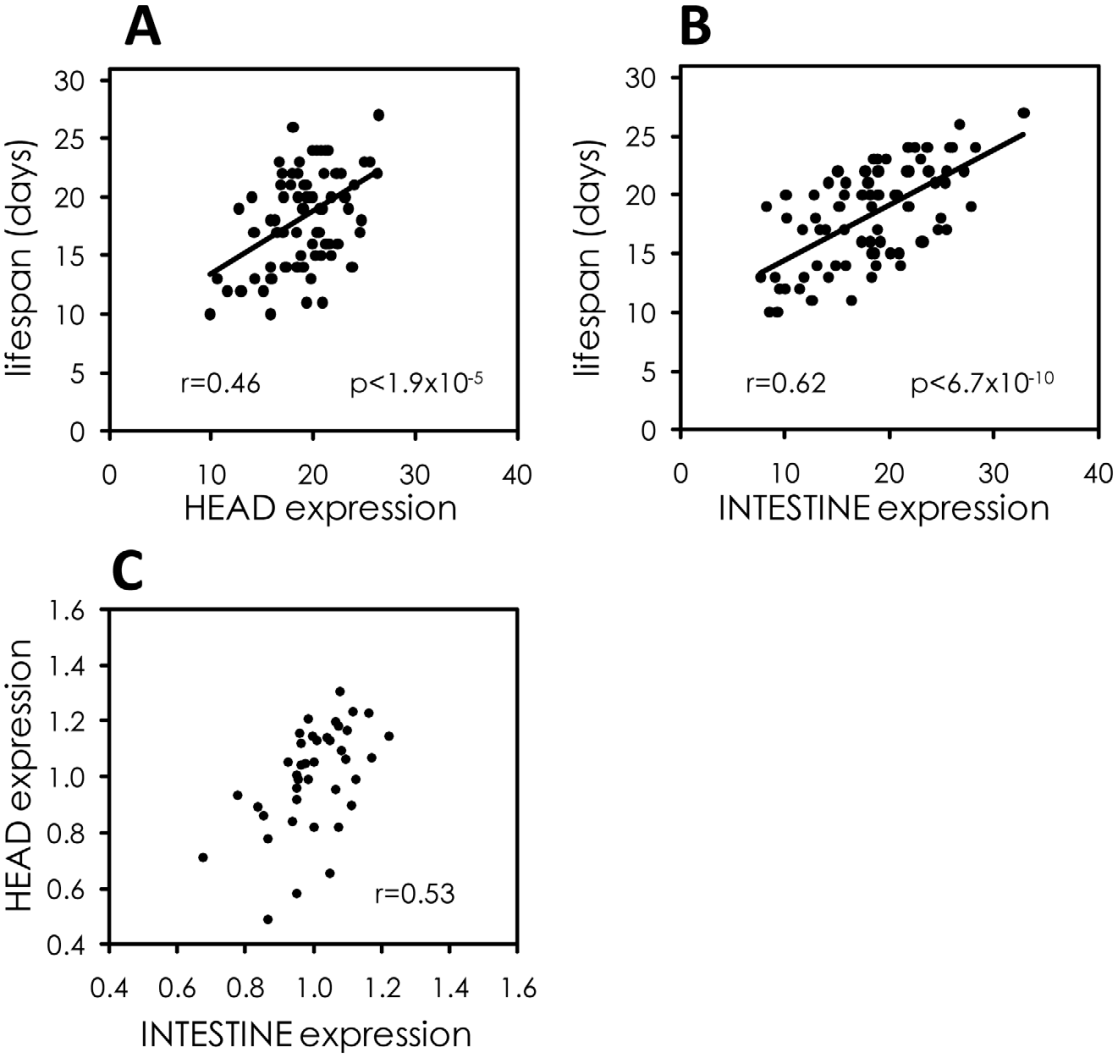
The intestine is a major tissue determining variable individual lifespan. (A) Scatterplot comparing expression of *sod-3::mCherry* in the head at day 9 of adulthood to remaining lifespan. Data are from 78 *sod-3::mCherry* expressing worms. Pearson correlation and p-value are shown. Expression levels are in arbitrary units. (B) Scatterplot comparing expression of *sod-3::mCherry* in the intestine at day 9 of adulthood to remaining lifespan. Data are from 78 *sod-3::mCherry* expressing worms. Expression levels are in arbitrary units. (C) Correlation between relative *sod-3* expression in the head and the intestine within individual *sod-3::mCherry* expressing worms at day 9 of adulthood (n = 40, r = 0.53).

*sod-3* is expressed throughout the worm, including many cells in the head as well as the intestine. The intestine is a primary site for response to pathogenic infection [8] and also for transcriptional regulation of *daf-16* FOXO [14]. Three results indicate that the intestine is a major tissue responsive to variable pathogenicity, which in turn determines individual lifespan. First, we measured mCherry fluorescence produced from the *sod-3* promoter separately in the head and the anterior intestine. Expression in the intestine showed a higher correlation with lifespan than did expression in the head, demonstrating that intestinal *sod-3* expression is the main contributor to the lifespan correlation (Figure 7A and 7B). Second, *E. coli* and *B. subtilis* had different effects on *sod-3* expression in the intestine, but similar effects on expression in the head throughout lifespan (Figure S9B and S9C). This result is consistent with the idea that differences in lifespan due to growth on *E. coli* or *B. subtilis* are due to effects in the intestine. Third, we observed that there were variable levels of *sod-3* expression in the head and intestine in individual worms of the same age (Figure S9A). However, *sod-3* expression in the head showed little correlation to expression in the intestine in individual worms (Figure 7C).

### Improved prediction of remaining lifespan from combinations of molecular markers

Expression from two markers could provide more information about remaining lifespan than one marker alone if, for example, expression of the markers were to vary independently from each other in individuals. We calculated the correlation between marker expression and remaining lifespan for *sod-3*, each of the seven other marker genes, and for each of the seven double combinations with *sod-3* (Table 2). For six genes (*ugt-9*, *unc-54*, *myo-3*, *pha-4*, C26B9.5 and *elt-3*), we found a higher correlation with remaining lifespan using these markers in combination with *sod-3* expression than using one marker alone (p<0.05 linear regression). The best combinations were *sod-3* expression combined with expression from *ugt-9*, C26B9.5 or *pha-4*, which had correlations with lifespan between 0.66 and 0.70, accounting between 43% and 49% of the variability in individual lifespan. As discussed above, the seventh gene is *daf-16*, which acts in the same pathway as *sod-3* and thus *daf-16* expression provides redundant information with *sod-3* expression about remaining lifespan in individual worms. As a control, we looked at the correlation between expression of *sod-3::GFP* and *sod-3::mCherry* in individual worms. As expected, we found that the combined GFP and mCherry expression levels did not improve the correlation with remaining lifespan compared to either *sod-3* marker alone (Table 2). These results show that a combination of aging markers can significantly improve the correlation with remaining lifespan in individual worms.

## Discussion

**Figure 8:**
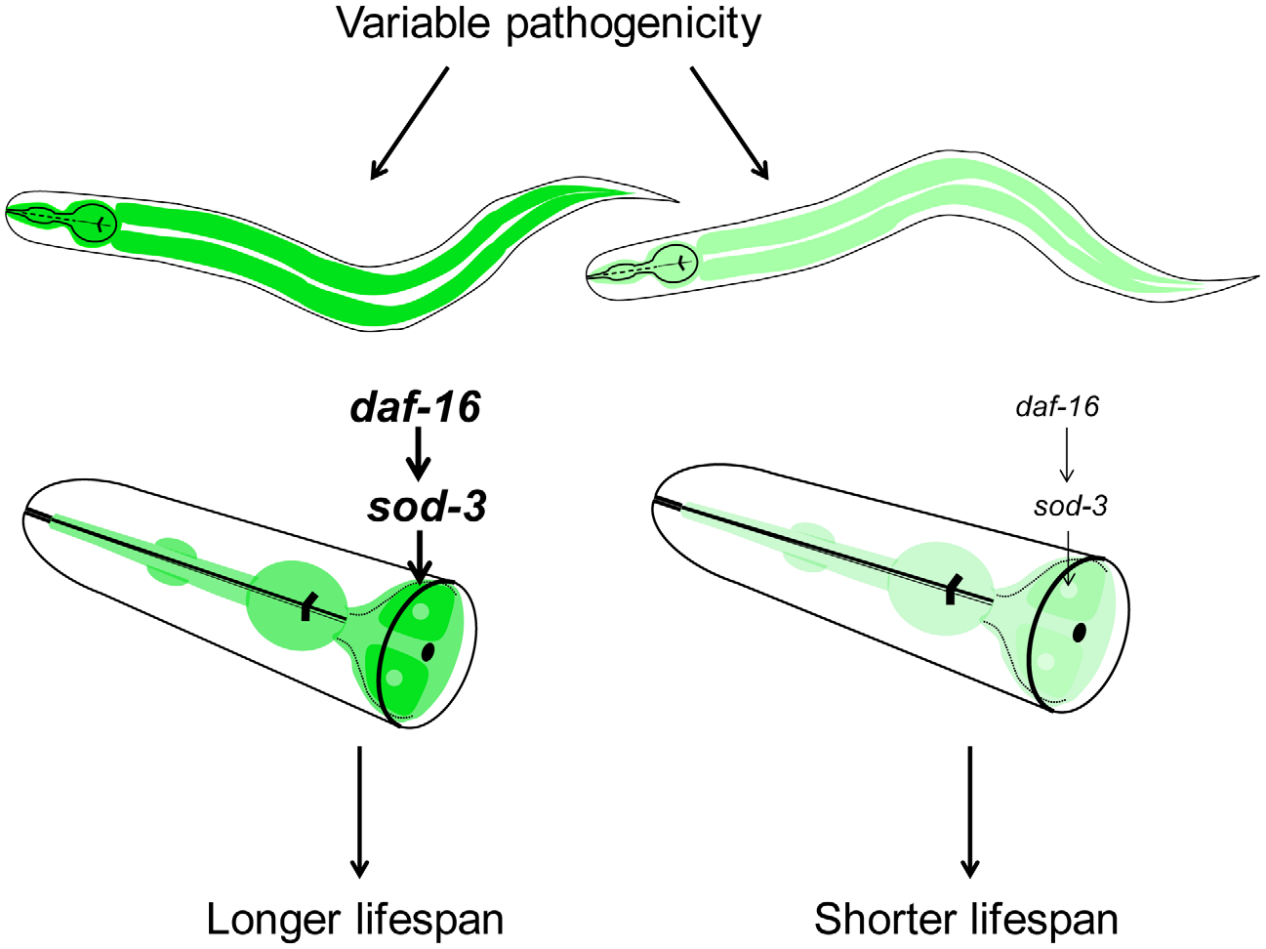
Model for lifespan variability in *C. elegans*. Variable pathogenicity in young adulthood leads to older or younger physiological age of individuals. Variable pathogenicity affects *daf-16* activity, which then regulates *sod-3* expression. Darker green and lighter green represent higher and lower *sod-3* expression in the intestine, respectively. Higher *sod-3* expression from worm to worm is correlative with longer individual lifespan.

This paper shows that a common cause of death for worms grown under normal lab conditions is pathogenicity from ingested food. We propose a model in which there is a variable effect of pathogenicity between individuals in an isogenic and chronologically identical population, resulting in differences in lifespan (Figure 8). *E. coli* is mildly pathogenic [18], [19], [20], and individuals may be affected to different extents when eating *E. coli* as food. The pathogenicity from the ingested bacteria primarily affects the intestine. Ingestion of *E. coli* activates *daf-16* FOXO activity via the insulin-like signaling pathway, which induces a beneficial stress response that is protective and extends lifespan. *sod-3* is a downstream target of DAF-16, and expression of *sod-3* indicates levels of DAF-16 activation. High levels of *sod-3* expression correspond to high levels of DAF-16 activation and longer lifespan, and *vice versa* for low levels of *sod-3* expression. Mutations in *sod-3* do not affect lifespan, indicating that *sod-3* activity is not in itself functionally important for lifespan but that *sod-3* expression is a marker for physiological age because it reports the level of activity of *daf-16* FOXO. This paper provides novel insights about a common cause of death for worms grown under normal laboratory conditions, and highlights the complex and interconnected roles of aging and disease in specifying lifespan.

Where does the variability in bacterial pathogenicity between individuals arise? One possibility is that the variability arises from extrinsic environmental differences. For instance, there might be variability in pathogenicity of the *E. coli* itself in different regions of the plate, which might affect worms to different extents depending on which region they occupy. However, this appears unlikely because worms can traverse all regions of a plate in a day, and because we see a correlation between *sod-3* expression and remaining lifespan even when the plates are specially prepared to have even lawns of *E. coli*. A more likely possibility is that the variability might be intrinsic to the worm itself. Different worms might express different levels of DAF-16 stemming from intrinsic differences such as noise in the expression machinery. When confronted by mildly pathogenic *E. coli*, some worms could mount a stronger protective response than others, giving rise to correlated differences in *sod-3* expression and variability in lifespan.

Evidence that bacterial pathogenicity in the intestine is a major source of stochasticity in physiological aging in individual worms comes from comparing the effects caused by feeding worms live *E. coli* versus three other food sources (*B. subtilis*, UV-killed *E. coli*, and *C. crescentus*). The most obvious differences between these foods is that live *E. coli* is mildly pathogenic whereas the others are either non-pathogenic or have less pathogenicity. It seems unlikely that the effects on lifespan seen with UV-killed *E. coli*, *B. subtilis* and *C. crescentus* are due to caloric restriction stemming from difficulty in ingesting these foods. Worms fed these three bacteria appear normal in size rather than thin, develop normally and produce a normal brood size [19], indicating that these worms are not dietary restricted.

What happens when worms are switched from *E. coli* as a food source (mildly pathogenic) to non-pathogenic bacteria such as *B. subtilis*? According to the model, worms fed *E. coli* experience variable levels of pathogenicity whereas this variability is reduced or absent for worms fed *B. subtilis*, resulting in four measurable differences. First, worms have a longer lifespan on non-pathogenic bacteria (Figure S4). Second, since *sod-3* expression is induced by pathogenicity, expression is lower on non-pathogenic bacteria compared to *E. coli* (Figure S9B and S9C). Third, the variable pathogenicity arising from growth on *E. coli* generates more fluctuation in *sod-3* expression compared to growth on *B. subtilis* (Figure 4). Fourth, variable pathogenicity from growth on *E. coli* results in a correlation between expression of *sod-3* and remaining lifespan of individual worms (Figure 3). Finally, when worms are grown on *B. subtilis*, they die at a later time and the cause of death is currently unknown. The cause of death for *B. subtilis*-fed worms could have more, less or similar variability between individuals compared to death from *E. coli*-derived pathogenicity. Hence, lifespan of worms fed *B. subtilis* could show more, less or the same amount of variability as worms fed *E. coli*, depending on the cause of death. In fact, we found that the variability of lifespan of worms grown on *B. subtilis* is 20% less than that of *E. coli*-fed worms (A.S.B., unpublished observations).

Our results identify a number of fluorescent reporters that can be used as markers of physiological age. Previously, the most common way to determine the rates of aging in *C. elegans* was to measure the lifespan of a population of worms. The aging markers presented in this paper will be useful tools to study aging because one can measure the age of individual worms and because expression analysis of fluorescent markers in individual worms is less time-consuming than lifespan analysis of a population of worms. *hsp-16::GFP* has been previously used as a marker for physiological age [13], but this marker requires heat shock treatment in order to predict remaining lifespan and this treatment prolongs lifespan in itself. Additionally, *drosomycin*, *hsp22* and *hsp70* are partially predictive of remaining lifespan in *Drosophila* [26], [27].

In addition to *sod-3*, we identified seven other aging biomarkers that could provide information about mechanisms responsible for variability in aging. For six of the biomarkers (*ugt-9*, *pha-4*, *myo-3*, *unc-54*, *C26B9.5*, and *elt-3*), combinations of *sod-3* and a second marker provide better prediction of remaining lifespan than just one of the markers alone. This result suggests that the different markers may be responsive to aging pathways that are distinct from those controlling *sod-3* expression. For example, age-related decrease in expression of the GATA transcription factor ELT-3 is caused by drift of the upstream regulatory network that controls ELT-3 expression [10]. Thus, simultaneous measurement of *sod-3* and *elt-3* expression in a worm would show the status of two aging pathways (pathogenic induction and developmental drift) in that individual, and therefore provide better information about its physiological age and remaining lifespan. For the remaining five aging biomarkers, DNA microarray experiments indicate that they are not regulated by *daf-16* FOXO [10], [28]. Future studies may reveal the source of variation governing the other aging markers described in this paper, which will illuminate other mechanisms that limit lifespan of worms grown under normal lab conditions.

## Materials and Methods

### Bacterial growth

UV-killed *E. coli* was prepared as described [8]. *C. crescentus* was grown as described [29]. Since *C. crescentus* does not grow on NGM plates, plates were seeded with 300 µl of resuspended *C. crescentus*, allowed to dry, and then subjected to UV irradiation as described [8].

### *C. elegans* fluorescent reporters

We used three *sod-3* fluorescent reporters. The first is a multi-copy insertion reporter that expresses cytoplasmic GFP from the *sod-3* promoter (referred to as *sod-3::GFP*) [14]. The second is a low-copy insertion reporter that expresses histone H2B fused to mCherry from the *sod-3* promoter (referred to as *sod-3::mCherry*) [30]. The third is a multi-copy extrachromosomal array reporter that expresses histone H2B fused to GFP from the *sod-3* promoter (referred to as extrachromosomal *sod-3::GFP*) [10].

*daf-16::GFP* is a multi-copy integrated translational reporter that expresses GFP at the C terminus of the DAF-16 protein [24]. *myo-3::GFP* is an multi-copy integrated reporter that expresses nuclear-targeted GFP–LacZ from the *myo-3* promoter [31]. *unc-54::mCherry* and *pha-4::mCherry* are low-copy integrated reporters that express histone H1 fused to mCherry from the *unc-54* or the *pha-4* promoter [30]. *ugt-9::GFP* and *elt-3::GFP* are multi-copy extrachromosomal array reporters that expresses histone H2B fused to GFP from the *ugt-9* or the *elt-3* promoter [10].

Promoter::H1::mCherry constructs for *C26B9.5* and *mif-2* were made using promoters from the Vidal lab promoterome [32] in combination with a modified Gateway cloning system [33] vector (pD4H1cherry [30]). Transgenic lines with integrated copies of the reporter were made by microparticle bombardment [34].

### Lifespan prediction assay

Approximately 80 age-synchronized worms were transferred to 1 mM aldicarb-NGM plates for 2–3 hours to induce paralysis [35]. Worms were then individually transferred to FUDR plates. Each worm was then photographed using 20× lens. Images were analyzed using ImageJ. For any given comparison, all pictures were taken on the same day with the same microscope settings. Each plate containing a single worm was labeled with the corresponding picture number and plates were scored for dead worms daily.

Lifespan analyses were conducted at 20°C as previously described [36]. Age refers to days following adulthood, and p values were calculated using the log-rank (Mantel-Cox) method. Individuals were excluded from the analysis when their gonad was extruded, or when they desiccated by crawling onto the edge of the housing plate.

### Lipofuscin autofluorescence quantification

Worm gut autofluorescence was imaged using a 525 nm bandpass filter. A Zeiss Axioplan microscope equipped with Zeiss AxioVision 4.6 software was used for quantitative fluorescence microscopy. Images were captured with 10× lens and analyzed using ImageJ. Gut autofluorescence time courses were done using at least 15 worms per age. All pictures were taken on the same day with the same microscope settings.

### Statistical analyses

To test remaining lifespan prediction of the age-related slope in the longitudinal *sod-3::mCherry* expression experiment, linear regression analysis was used with the following model:
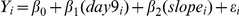

*Y_i_* is the lifespan of worm *i*, *day* 9*_i_* is the fluorescence of worm *i* at day 9, *slope_i_* is the slope of fluorescence by age for worm *i* and *ε_i_* is a random error term. The coefficients were estimated by least squares from the data. Expression at day 9 (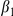 term) was statistically significant (p<3.0×10^−7^). Age-related slope of *sod-3* expression (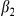 term) was not statistically significant (p>0.21).

To test the combined effect of multiple measurements within single worms, multiple regression analysis was used with the following model:
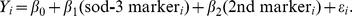


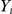
 is the lifespan of worm *i*, sod-3 marker and 2nd marker are the *sod-3* and additional marker expression levels respectively, 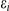 is a random error term, and 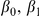 and 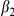 are regression coefficients. Likelihood ratio *F*-tests were used to determine the significance of 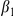 and 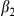. For example, to test whether addition of a *daf-16::GFP* second marker adds significant information about lifespan prediction compared to using *sod-3::mCherry* alone, we test the hypothesis that 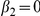. This is done by comparing the following two predictions of lifespan:

Model (1): 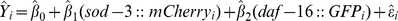

Model (2): 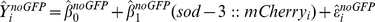.

*Y* is the original matrix of measured lifespans, 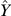
is the matrix of predicted lifespans from the full model (1), and 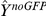 is the matrix of predicted lifespans from the partial model (2) that leaves out the *daf-16::GFP* term. With *n* independent worms, the likelihood ratio test compares the residual sum of squares of the two models and rejects the hypothesis that 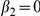 if 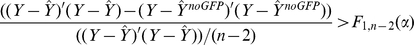, where 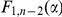 is the cumulative distribution of an *F*-distribution with 1 and *n*-2 degrees of freedom.

Using this test, we determined that addition of the *daf-16::GFP* expression term did not significantly improve the model compared to using *sod-3::mCherry* alone (*p*-value>0.2, and so we cannot reject the hypothesis that 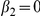). A similar approach showed that adding *sod-3::mCherry* expression significantly improved a model using *daf-16::GFP* expression alone (*p*-value<0.001, so we can reject the hypothesis that 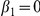).

### Worm transgenic lines

The *sod-3* gene and regulatory regions (2.57 kbp fragment) were amplified by PCR from wild type N2 worm genomic DNA (forward primer: 5′ ATT CGC AGA AAA AAG TCG TTG C 3′, reverse primer: 5′ TTT CAG TGT ACC GAG TGA AGT TC 3′). The *sod-3* PCR fragment was cloned into the TOPO pCR 2.1 vector (Invitrogen). TOPO pCR 2.1 plasmid with the coding *sod-3* region was coinjected at different concentrations (5 ng/µl and 40 ng/µl) with *pha-1* encoding plasmid into *pha-1* mutant worms to generate extrachromosomal array *sod-3* overexpressing lines. A control line was generated by injecting *pha-1* encoding plasmid alone into *pha-1* mutant worms.

## Supporting Information

Figure S1 Fluorescent marker expression during normal aging in adult hermaphrodites. Expression is from *daf-16::GFP*, *myo-3::GFP*, *unc-54::mCherry*, *pha-4::mCherry* and *C29B9.5::mCherry* (see materials and methods). Lifopuscin pigment was measured by gut autofluorescence. y-axis shows levels of expression in arbitrary units. x-axis shows age of worms. Bars indicate S.E.M. n = 15 or greater in each time point.
(PDF)

Figure S2 Longitudinal *sod-3::mCherry* expression of individual worms during aging. y-axis indicates expression level in arbitrary units. x-axis indicates days of adulthood. Every colored line represents *sod-3::mCherry* expression of an individual worm during aging.
(PDF)

Figure S3 Lifespan curves for transgenic worms with extra copies of wild-type *sod-3*. All lifespans were done at 20°C. y-axis indicates % of worms that are alive. x-axis indicates day of adulthood. (A) Transgenic worms were injected with the *sod-3* transgene at 5 ng/µl concentration (low *sod-3* copy number). (B) Transgenic worms were injected with the *sod-3* transgene at 40 ng/µl concentration (high *sod-3* copy number).
(PDF)

Figure S4 Lifespan curves for adult hermaphrodite worms maintained on live *E. coli*, UV-killed *E. coli*, *C. crescentus* and *B. subtilis* at 20°C. y-axis indicates % of worms that are alive. x-axis indicates day of adulthood.
(PDF)

Figure S5 Correlation between *sod-3* expression and remaining lifespan for worms fed different types of bacteria. Shown are scatterplots comparing expression of *sod-3*::*GFP* in middle-aged worms to their remaining lifespan. x-axis shows expression levels in arbitrary units. y-axis shows lifespan in days. (A) *sod-3::GFP* expression in worms (n = 77) grown on plates with live *E. coli*. *sod-3::GFP* expression was measured at day 8 of adulthood (49% of their mean lifespan). (B) *sod-3::GFP* expression from worms (n = 75) maintained on UV-killed *E. coli*. *sod-3::GFP* expression was measured at day 9 of adulthood (47% of their mean lifespan). (C) *sod-3::GFP* expression from worms (n = 78) maintained on *B. subtilis*. *sod-3::GFP* expression was measured at day 12 of adulthood (54% of their mean lifespan). (D) *sod-3::GFP* expression from worms (n = 80) maintained on *C. crescentus*. *sod-3*::*GFP* expression was measured at day 12 of adulthood (59% of their mean lifespan).
(PDF)

Figure S6 Scatterplot showing correlation between *sod-3::GFP* expression and remaining lifespan in 8 day old *daf-2(e1370)*;*daf-16(mu86)* worms (n = 85). x-axis shows expression levels in arbitrary units. y-axis shows lifespan in days. The Pearson correlation between expression and remaining lifespan, and p-value are shown in the plot.
(PDF)

Figure S7 Comparison of *sod-3*::GFP expression for *daf-16(+);elt-3(+)* worms at day 9 of adulthood, *elt-3(vp1)* at day 8 of adulthood, and *daf-2(e1370);daf-16(mu86)* mutants at day 8 of adulthood. Bars indicate S.E.M. Expression was measured in the anterior portion of the worm and is represented in arbitrary units.
(PDF)

Figure S8 *daf-16::*GFP expression during aging in adult hermaphrodites fed either *E. coli* or *B. subtilis*. y-axis shows levels of expression in arbitrary units. x-axis shows age of worms. Bars indicate S.E.M. n = 30–40 worms in each time point.
(PDF)

Figure S9 (A) *sod-3* expression in the head versus the intestine in three individual hermaphrodites at day 8 of adulthood. Images show examples of variation in expression for *sod-3::GFP* in the head and intestine. (B) intestinal *sod-3::mCherry* expression during aging in worms maintained in *E. coli* (n = 20–40) or *B. subtilis* (n = 20–40). y-axis indicates expression level in arbitrary units. x-axis indicates days of adulthood. Bars indicate S.E.M. (C) Head *sod-3::mCherry* expression during aging of worms maintained in *E. coli* (n = 20–40) or *B. subtilis* (n = 20–40).
(PDF)

Table S1 *sod-3* expression and lifespan correlation at different ages. Experiment #1 was performed using *sod-3::mCherry* worms. Experiment #2 was performed using *sod-3::GFP* worms.
(PDF)

Table S2 Variability in *sod-3* abundance for worms fed *E. coli* and *B. subtilis*.
(PDF)

Table S3 Lifespan differences for worms maintained on one type of bacteria (*E. coli* or *B. subtilis*) and then shifted to the other bacteria at day 8. All lifespans were done at 20°C.
(PDF)
