# Supplementary figures and images for: Variable Pathogenicity Determines Individual Lifespan in Caenorhabditis elegans
Source: PLoS Genet. 2011 Apr 14;7(4):e1002047. doi: 10.1371/journal.pgen.1002047 (PMC3077391; doi:10.1371/journal.pgen.1002047)

gene name

expression

*daf-16*

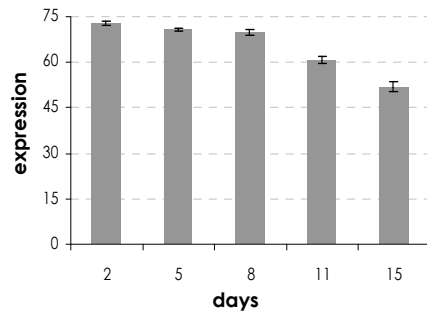

*myo-3*

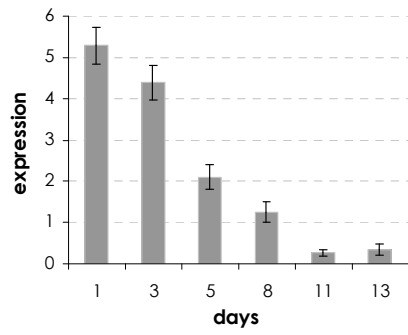

*unc-54*

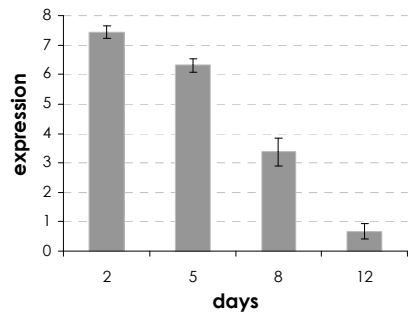

gene name

expression

*pha-4*

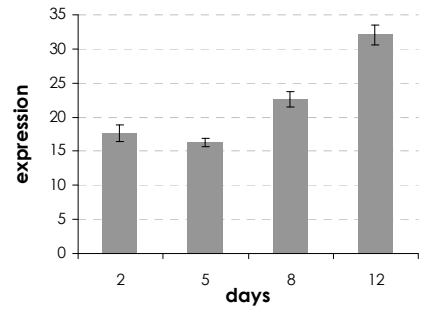

*C26B9.5*

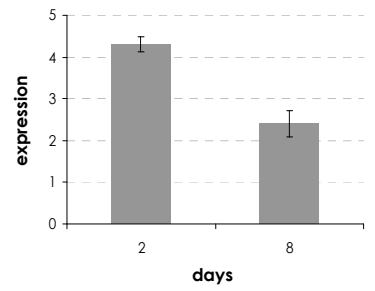

lipofuscin

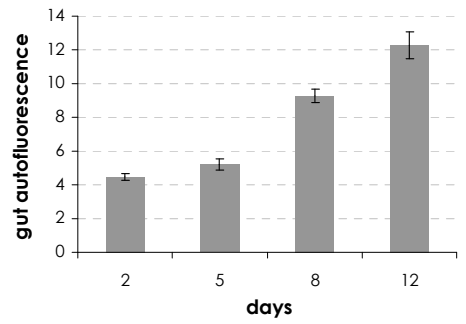

Supplement: Figure S1 — Fluorescent marker expression during normal aging in adult hermaphrodites. Expression is from daf-16::GFP, myo-3::GFP, unc-54::mCherry, pha-4::mCherry and C29B9.5::mCherry (see materials and methods). Lifopuscin pigment was measured by gut autofluorescence. y-axis shows levels of expression in arbitrary units. x-axis shows age of worms. Bars indicate S.E.M. n = 15 or greater in each time point. (PDF) [file pgen.1002047.s001.pdf]

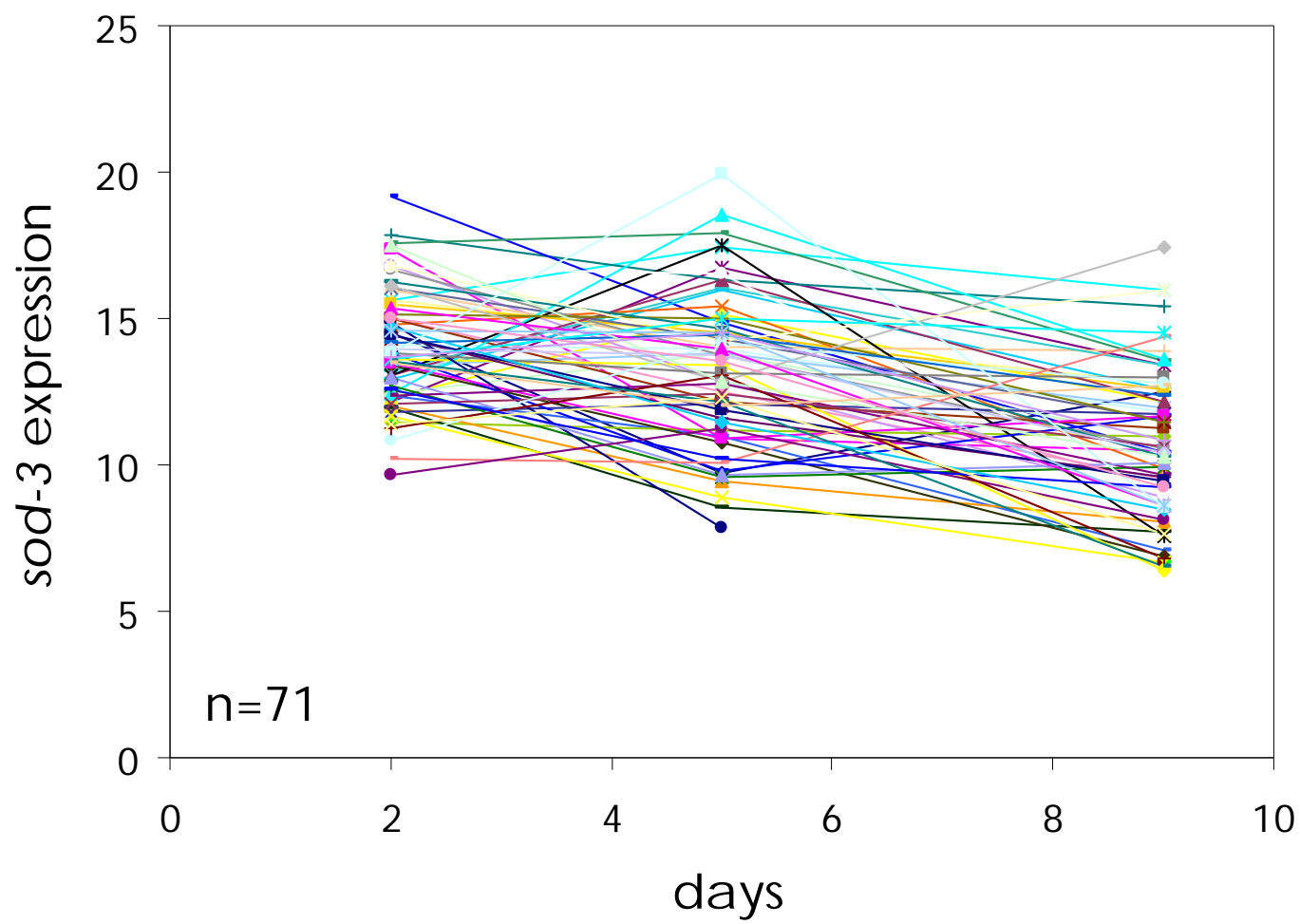

Supplement: Figure S2 — Longitudinal sod-3::mCherry expression of individual worms during aging. y-axis indicates expression level in arbitrary units. x-axis indicates days of adulthood. Every colored line represents sod-3::mCherry expression of an individual worm during aging. (PDF) [file pgen.1002047.s002.pdf]

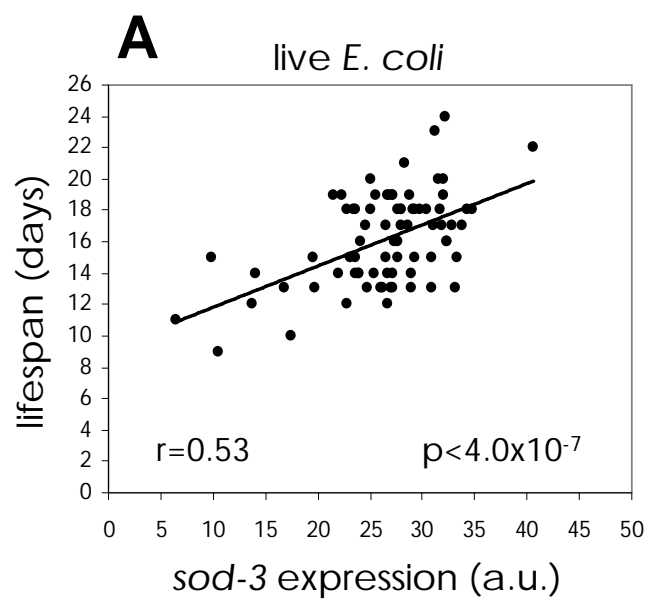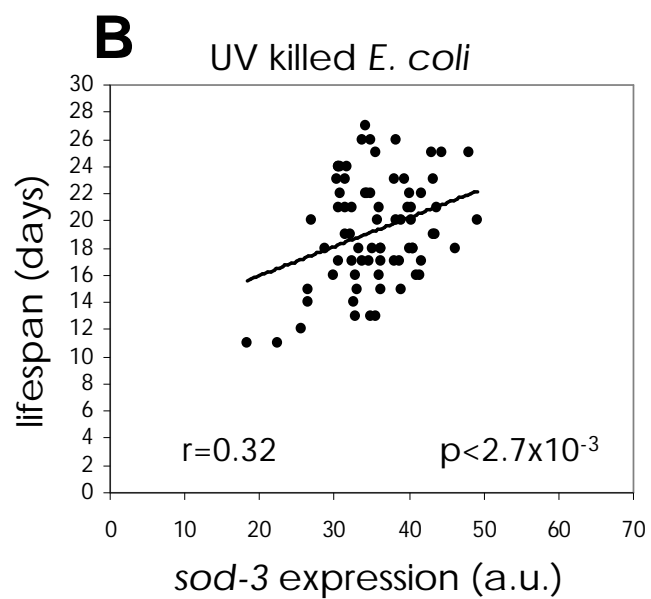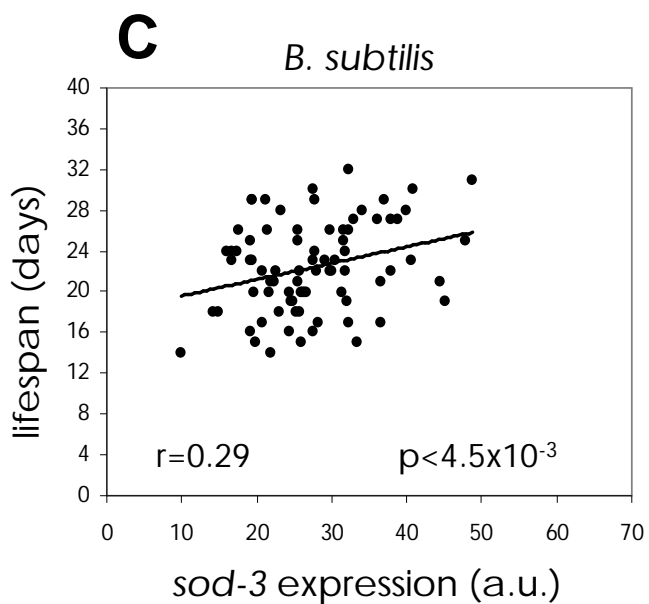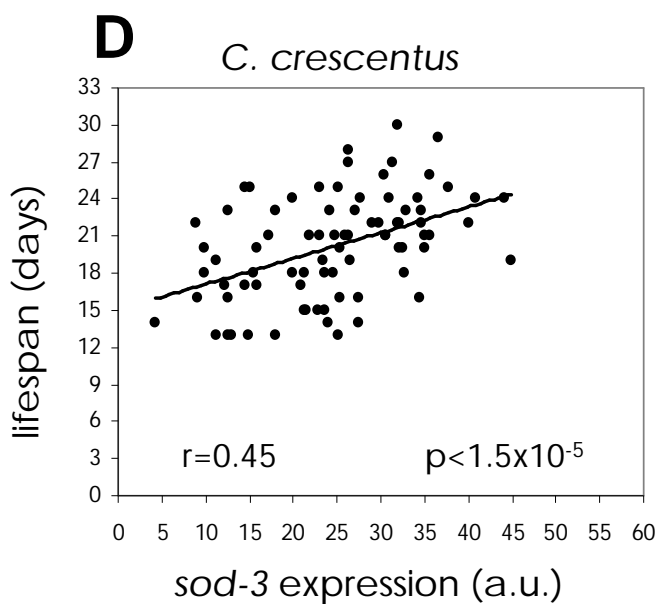

Supplement: Figure S5 — Correlation between sod-3 expression and remaining lifespan for worms fed different types of bacteria. Shown are scatterplots comparing expression of sod-3::GFP in middle-aged worms to their remaining lifespan. x-axis shows expression levels in arbitrary units. y-axis shows lifespan in days. (A) sod-3::GFP expression in worms (n = 77) grown on plates with live E. coli. sod-3::GFP expression was measured at day 8 of adulthood (49% of their mean lifespan). (B) sod-3::GFP expression from worms (n = 75) maintained on UV-killed E. coli. sod-3::GFP expression was measured at day 9 of adulthood (47% of their mean lifespan). (C) sod-3::GFP expression from worms (n = 78) maintained on B. subtilis. sod-3::GFP expression was measured at day 12 of adulthood (54% of their mean lifespan). (D) sod-3::GFP expression from worms (n = 80) maintained on C. crescentus. sod-3::GFP expression was measured at day 12 of adulthood (59% of their mean lifespan). (PDF) [file pgen.1002047.s005.pdf]

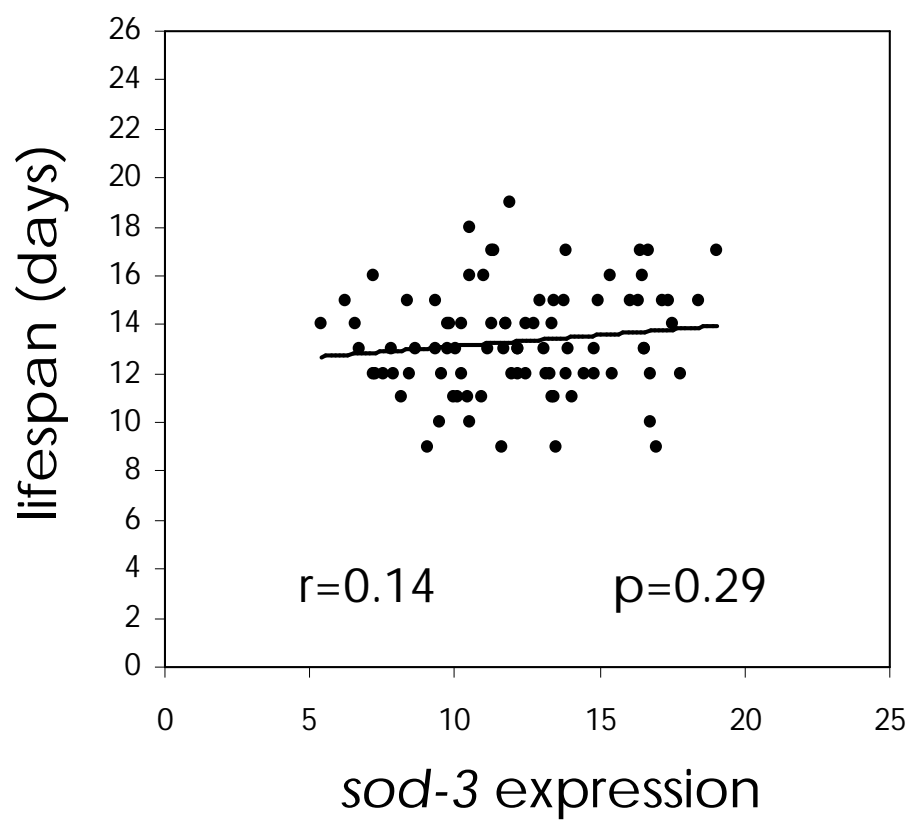

Supplement: Figure S6 — Scatterplot showing correlation between sod-3::GFP expression and remaining lifespan in 8 day old daf-2(e1370);daf-16(mu86) worms (n = 85). x-axis shows expression levels in arbitrary units. y-axis shows lifespan in days. The Pearson correlation between expression and remaining lifespan, and p-value are shown in the plot. (PDF) [file pgen.1002047.s006.pdf]

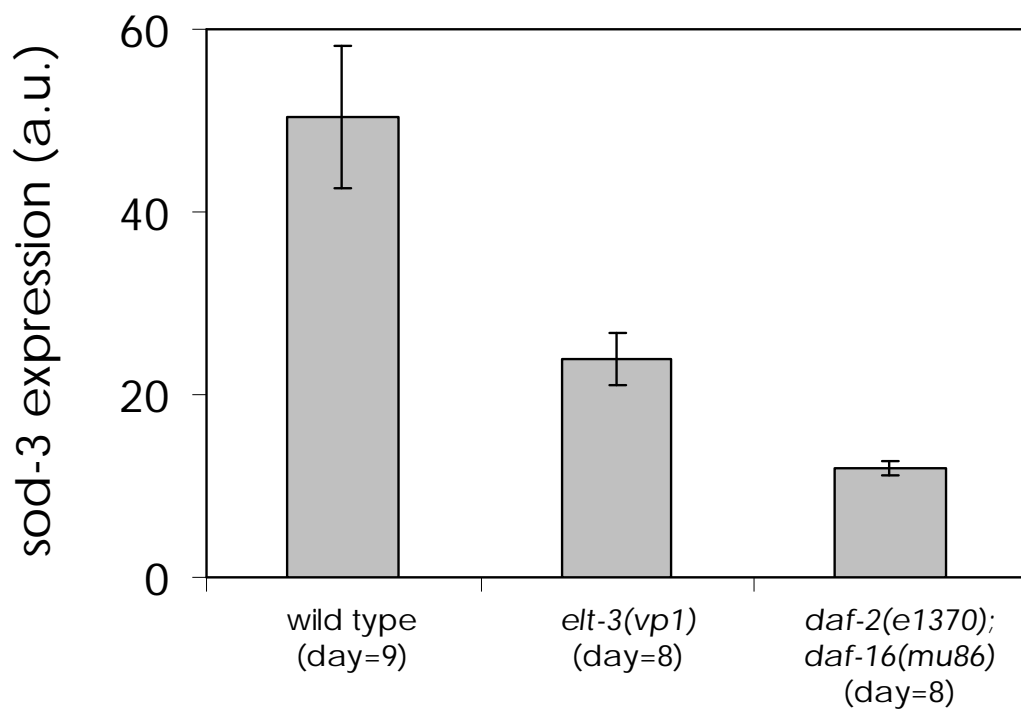

Supplement: Figure S7 — Comparison of sod-3::GFP expression for daf-16(+);elt-3(+) worms at day 9 of adulthood, elt-3(vp1) at day 8 of adulthood, and daf-2(e1370);daf-16(mu86) mutants at day 8 of adulthood. Bars indicate S.E.M. Expression was measured in the anterior portion of the worm and is represented in arbitrary units. (PDF) [file pgen.1002047.s007.pdf]

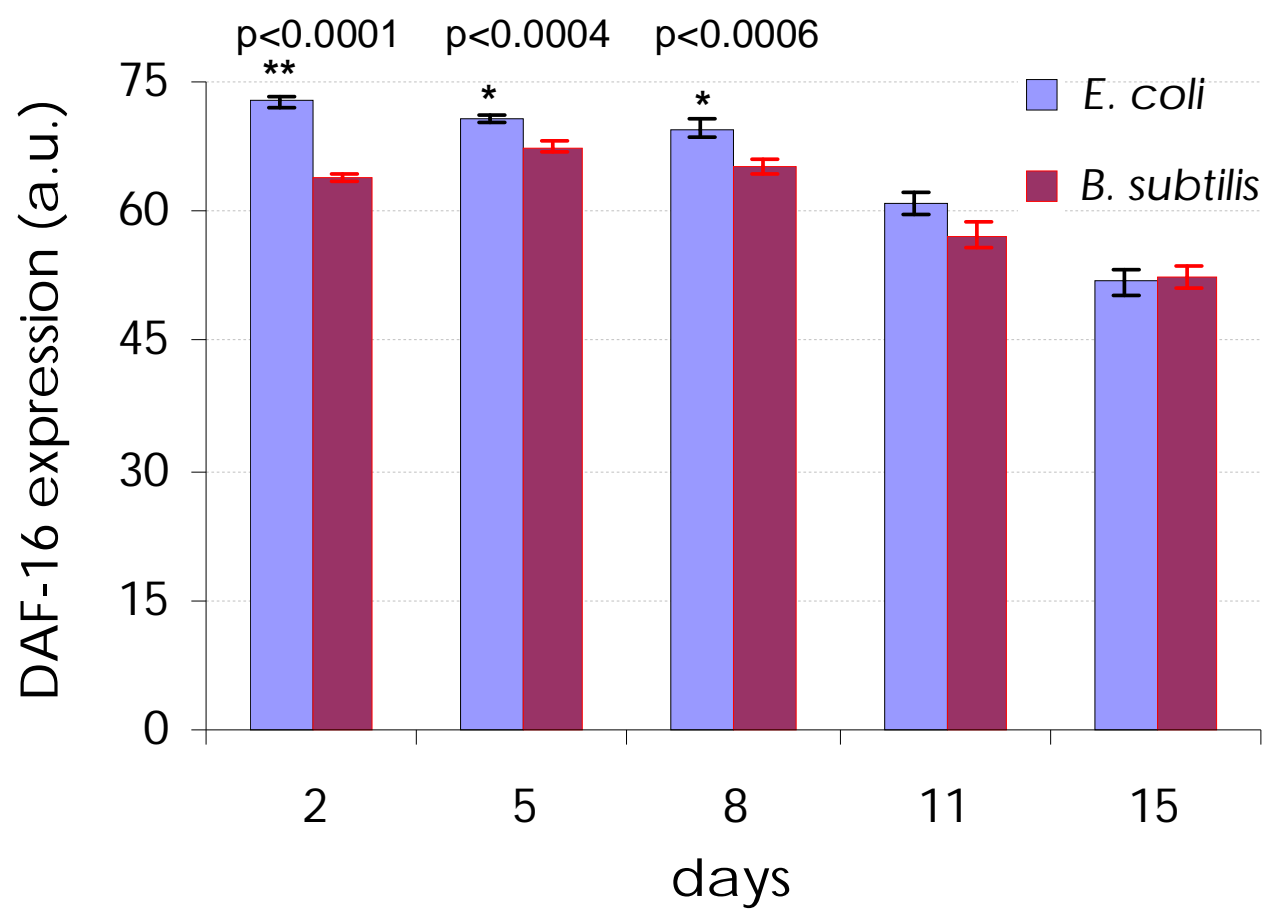

Supplement: Figure S8 — daf-16::GFP expression during aging in adult hermaphrodites fed either E. coli or B. subtilis. y-axis shows levels of expression in arbitrary units. x-axis shows age of worms. Bars indicate S.E.M. n = 30–40 worms in each time point. (PDF) [file pgen.1002047.s008.pdf]
