# Supplementary material for: Variable Pathogenicity Determines Individual Lifespan in Caenorhabditis elegans
Source: PLoS Genet. 2011 Apr 14;7(4):e1002047. doi: 10.1371/journal.pgen.1002047 (PMC3077391; doi:10.1371/journal.pgen.1002047)
Supplement: Figure S3 — Lifespan curves for transgenic worms with extra copies of wild-type sod-3. All lifespans were done at 20°C. y-axis indicates % of worms that are alive. x-axis indicates day of adulthood. (A) Transgenic worms were injected with the sod-3 transgene at 5 ng/µl concentration (low sod-3 copy number). (B) Transgenic worms were injected with the sod-3 transgene at 40 ng/µl concentration (high sod-3 copy number). (PDF) [file pgen.1002047.s003.pdf]

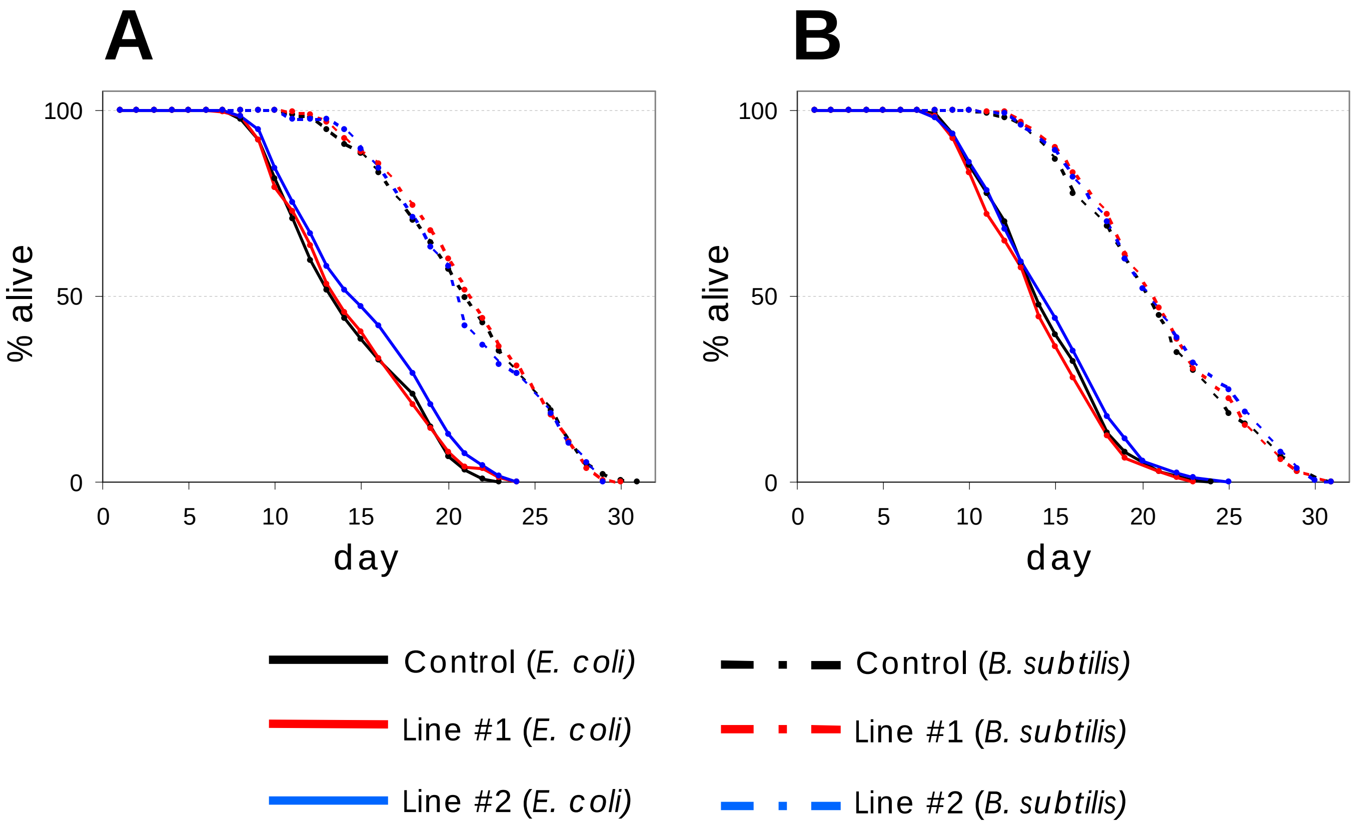

	sod-3 plasmid concentration 5 ng/µl						*sod-3* plasmid concentration 40 ng/µl					
	E. coli			*B. subtilis*			*E. coli*			*B. subtilis*		
	control	line #1	line #2	control	line #1	line #2	control	line #1	line #2	control	line #1	line #2
n	144	173	186	155	178	38	188	189	171	186	174	172
median lifespan	13.2	13.4	14.3	21.0	21.2	20.5	13.8	13.6	14.2	20.3	20.4	20.6
lifespan difference		1.5%	8.3%		1.0%	-2.4%		-1.5%	2.9%		0.5%	1.5%
p value (log rank)		>0.05	>0.05		>0.05	>0.05		>0.05	>0.05		>0.05	>0.05
