# Supplementary material for: Variable Pathogenicity Determines Individual Lifespan in Caenorhabditis elegans
Source: PLoS Genet. 2011 Apr 14;7(4):e1002047. doi: 10.1371/journal.pgen.1002047 (PMC3077391; doi:10.1371/journal.pgen.1002047)
Supplement: Figure S4 — Lifespan curves for adult hermaphrodite worms maintained on live E. coli, UV-killed E. coli, C. crescentus and B. subtilis at 20°C. y-axis indicates % of worms that are alive. x-axis indicates day of adulthood. (PDF) [file pgen.1002047.s004.pdf]

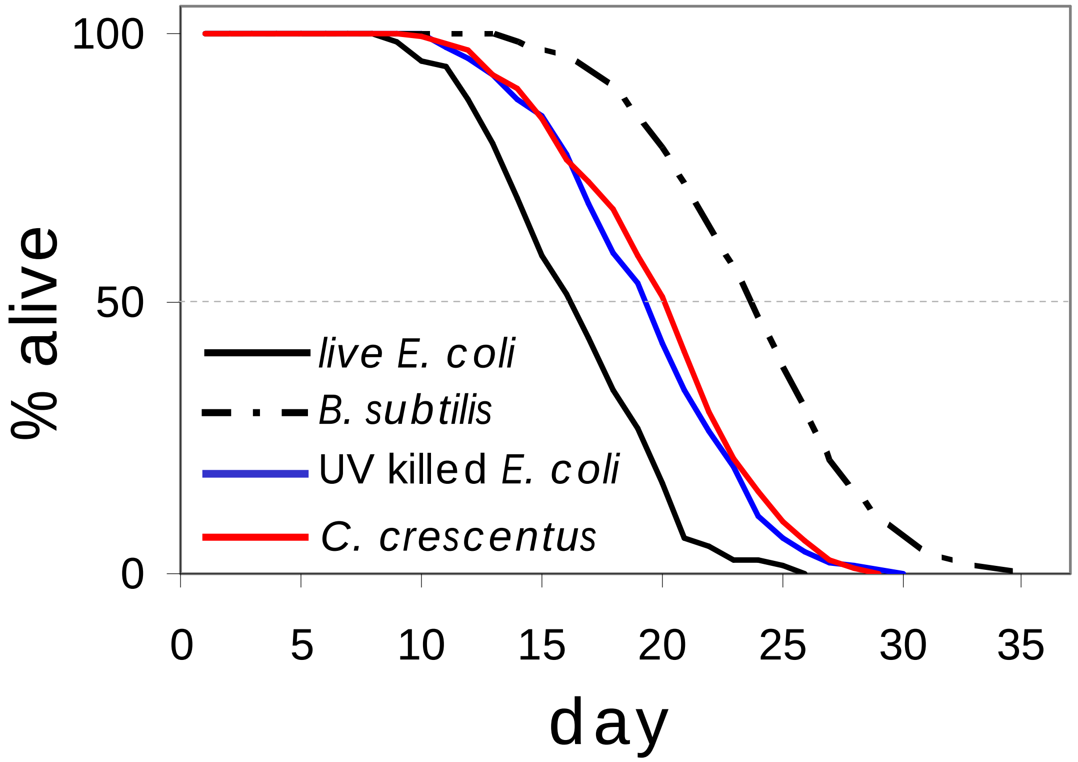

	live *E. coli*	UV killed *E. coli*	*C. crescentus*	*B. subtilis*
n	207	232	145	116
median lifespan (days)	16.3	19.3	20.0	23.9
median lifespan difference*		18.4%	22.7%	46.6%
p. value (log. rank)*		<0.001	<0.001	<0.001

* with respect to live *E. coli*
