# Supplementary material for: Variable Pathogenicity Determines Individual Lifespan in Caenorhabditis elegans
Source: PLoS Genet. 2011 Apr 14;7(4):e1002047. doi: 10.1371/journal.pgen.1002047 (PMC3077391; doi:10.1371/journal.pgen.1002047)
Supplement: Figure S9 — (A) sod-3 expression in the head versus the intestine in three individual hermaphrodites at day 8 of adulthood. Images show examples of variation in expression for sod-3::GFP in the head and intestine. (B) intestinal sod-3::mCherry expression during aging in worms maintained in E. coli (n = 20–40) or B. subtilis (n = 20–40). y-axis indicates expression level in arbitrary units. x-axis indicates days of adulthood. Bars indicate S.E.M. (C) Head sod-3::mCherry expression during aging of worms maintained in E. coli (n = 20–40) or B. subtilis (n = 20–40). (PDF) [file pgen.1002047.s009.pdf]

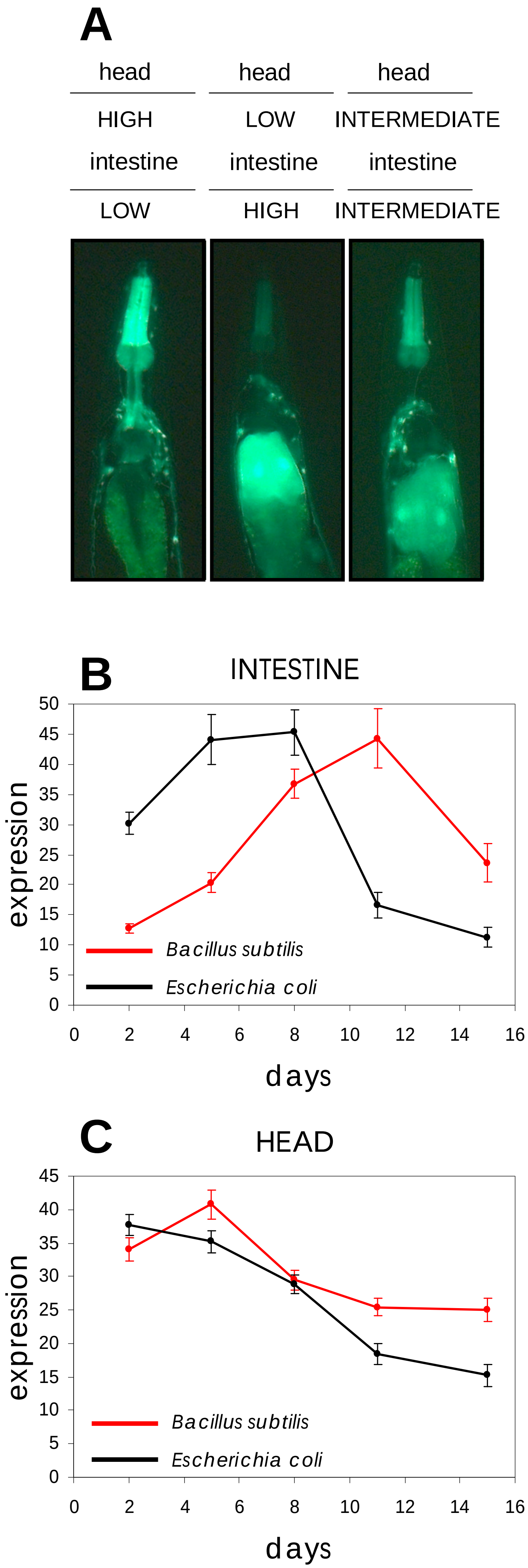
A
head
HIGH
intestine
LOW
head
LOW
intestine
HIGH
head
INTERMEDIATE
intestine
INTERMEDIATE
B
INTESTINE
expression
days
Bacillus subtilis
Escherichia coli
C
HEAD
expression
days
Bacillus subtilis
Escherichia coli
