# Supplementary material for: Variable Pathogenicity Determines Individual Lifespan in Caenorhabditis elegans
Source: PLoS Genet. 2011 Apr 14;7(4):e1002047. doi: 10.1371/journal.pgen.1002047 (PMC3077391; doi:10.1371/journal.pgen.1002047)
Supplement: Table S1 — sod-3 expression and lifespan correlation at different ages. Experiment #1 was performed using sod-3::mCherry worms. Experiment #2 was performed using sod-3::GFP worms. (PDF) [file pgen.1002047.s010.pdf]

	Experiment #1 (n=71)			Experiment #2 (n=69)		
	**day=2**	**day=5**	**day=9**	**day=2**	**day=5**	**day=9**
sod-3 expression and lifespan correlation (r)[a]	0.23	0.34	0.55	0.02	0.32	0.52
p value [b]	0.02	$1.7x10^{-3}$	$3.0x10^{-7}$	0.43	$4.2x10^{-3}$	$4.4x10^{-6}$
sod-3 expression variability [c]	0.14	0.19	0.24	0.21	0.22	0.42

[a] Pearson correlation coefficient between *sod-3* expression and lifespan

[b] One tailed p-value calculated for correlation significance

[c] Expression variability is defined as the standard deviation divided by the average expression
