# Supplementary material for: Variable Pathogenicity Determines Individual Lifespan in Caenorhabditis elegans
Source: PLoS Genet. 2011 Apr 14;7(4):e1002047. doi: 10.1371/journal.pgen.1002047 (PMC3077391; doi:10.1371/journal.pgen.1002047)
Supplement: Table S2 — Variability in sod-3 abundance for worms fed E. coli and B. subtilis. (PDF) [file pgen.1002047.s011.pdf]

	Variability in *sod-3* abundance (SD/mean)		
	day=8	day=12	day=14
E. coli	0.36	0.63	0.64
B. subtilis	0.21	0.24	0.28
