# Supplementary material for: Variable Pathogenicity Determines Individual Lifespan in Caenorhabditis elegans
Source: PLoS Genet. 2011 Apr 14;7(4):e1002047. doi: 10.1371/journal.pgen.1002047 (PMC3077391; doi:10.1371/journal.pgen.1002047)
Supplement: Table S3 — Lifespan differences for worms maintained on one type of bacteria (E. coli or B. subtilis) and then shifted to the other bacteria at day 8. All lifespans were done at 20°C. (PDF) [file pgen.1002047.s012.pdf]

	B. subtilis	*B. subtilis* → *E. coli*	*E. coli*	*E. coli* → *B. subtilis*
n	91	106	128	99
median lifespan	24.1	22.8	15.9	18.2
lifespan differences		-5.4% *		14.5% **
		25.3% ***		

* median lifespan difference with respect to worms kept solely on *B. subtilis* ($p<0.0025$ (log rank))

** median lifespan difference with respect to worms kept solely on *E. coli* ($p<0.0023$ (log rank))

*** median lifespan difference with respect to worms kept on *E. coli* and then shifted to *B. subtilis* at day 8 ($p<0.0001$ (log rank))
